# Smoking Is Associated with Shortened Airway Cilia

**DOI:** 10.1371/journal.pone.0008157

**Published:** 2009-12-16

**Authors:** Philip L. Leopold, Michael J. O'Mahony, X. Julie Lian, Ann E. Tilley, Ben-Gary Harvey, Ronald G. Crystal

**Affiliations:** 1 Department of Genetic Medicine, Weill Cornell Medical College, New York, New York, United States of America; 2 Division of Pulmonary and Critical Care Medicine, Weill Cornell Medical College, New York, New York, United States of America; University of Pittsburgh, United States of America

## Abstract

**Background:**

Whereas cilia damage and reduced cilia beat frequency have been implicated as causative of reduced mucociliary clearance in smokers, theoretically mucociliary clearance could also be affected by cilia length. Based on models of mucociliary clearance predicting that cilia length must exceed the 6–7 µm airway surface fluid depth to generate force in the mucus layer, we hypothesized that cilia height may be decreased in airway epithelium of normal smokers compared to nonsmokers.

**Methodology/Principal Findings:**

Cilia length in normal nonsmokers and smokers was evaluated in aldehyde-fixed, paraffin-embedded endobronchial biopsies, and air-dried and hydrated samples were brushed from human airway epithelium via fiberoptic bronchoscopy. In 28 endobronchial biopsies, healthy smoker cilia length was reduced by 15% compared to nonsmokers (p<0.05). In 39 air-dried samples of airway epithelial cells, smoker cilia length was reduced by 13% compared to nonsmokers (p<0.0001). Analysis of the length of individual, detached cilia in 27 samples showed that smoker cilia length was reduced by 9% compared to nonsmokers (p<0.05). Finally, in 16 fully hydrated, unfixed samples, smoker cilia length was reduced 7% compared to nonsmokers (p<0.05). Using genome-wide analysis of airway epithelial gene expression we identified 6 cilia-related genes whose expression levels were significantly reduced in healthy smokers compared to healthy nonsmokers.

**Conclusions/Significance:**

Models predict that a reduction in cilia length would reduce mucociliary clearance, suggesting that smoking-associated shorter airway epithelial cilia play a significant role in the pathogenesis of smoking-induced lung disease.

## Introduction

Cigarette smoke is composed of particulate matter, toxins, and oxidative chemicals, and poses a major stress on the airway epithelium [1]. Each puff of cigarette smoke contains >10^14^ oxidant molecules and >1000 xenobiotics [2], and exposure to cigarette smoke evokes significant biologic changes in the airway epithelium, even though many smokers are phenotypically normal [3]–[6]. One of the consequences of smoking is the associated reduction in mucociliary clearance, the process by which the coordinated action of cilia on the airway epithelium moves the airway surface fluid and mucus in a cephalad fashion, providing continuous cleansing of the airway surface [1], [7], [8]. In the context that mucociliary clearance is a vital defense against inhaled pathogens and particulates, a reduction in mucociliary clearance contributes to the increased susceptibility of cigarette smokers to respiratory tract infection, and to the increased risk for the development of chronic obstructive lung disease and bronchogenic carcinoma [9]–[13].

Although it is clear that smoking adversely affects mucociliary clearance, the mechanisms by which this occurs are not fully understood. One critical component is cilia, the hair-like projections on airway epithelial cells that move in metachronal waves and work in conjunction with mucus to clear the airway of inhaled particulates [9], [14]–[17]. Several reports demonstrate that cigarette smoke reduces ciliary beat frequency and interrupts the intercellular coordination of the metachronal waves [18]–[24], and ultrastructural studies have documented smoking-associated increased incidence of structural defects in cilia, including missing radial spokes, nexin links, central sheath, outer and inner dynein arms, and central microtubules, as well as more peripheral doublets and fused cilia [25]–[34].

As part of an ongoing study to assess the effect of smoking on gene expression in the airway epithelium using fiberoptic bronchoscopy and airway brushing to obtain samples of airway epithelium of phenotypic normal smokers and nonsmokers, we noted that the length of cilia on the epithelium of smokers appeared to be shorter than the cilia of nonsmokers. Shorter cilia could have profound functional consequences to host defense [35]. A review of the literature revealed only a few anecdotal reports of short cilia on the airway epithelium of patients with pulmonary disease [36]–[38]. The concept of bronchial cilia length in asymptomatic, healthy cigarette smokers has largely been ignored in the assessment of the consequences of smoking on the lung. Therefore, we initiated a study of cilia length in the airway epithelium of healthy smokers compared to healthy nonsmokers. Using airway epithelium obtained by biopsy and brushing, we assessed the average length of cilia in smokers compared to nonsmokers. Interestingly, using 4 different methods to prepare/assess samples, the data consistently demonstrate that length of airway epithelial cilia is reduced significantly in smokers compared to nonsmokers. Based on models of mucociliary clearance [15], [17], [35], [39], [40], the potential impact of shortened cilia may play a major role in the reduced mucociliary clearance observed in smokers, and may provide a novel target for therapeutic intervention.

## Methods

### Study Population

Normal, healthy smokers and nonsmokers were recruited by posting ads in local newspapers. The subjects were evaluated and tissue samples obtained in the Weill Cornell NIH General Clinical Research Center and the Department of Genetic Medicine Clinical Research Facility under an Institutional Review Board-approved clinical protocol. Before enrolling in the study, all subjects gave their informed written consent for the clinical evaluations and procedures. Individuals were determined to be phenotypically normal based on standard history, physical exam, complete blood count, coagulation studies, liver function tests, urine studies, chest X-ray, EKG, and pulmonary function tests. Overall, the study population consisted of a total of 75 individuals, 42 smokers and 33 nonsmokers (Table 1). All were phenotypic normals based on symptoms, physical exam, lung function and chest X-ray. The smoker group was confirmed to be current smokers by assessment of levels of urine nicotine and cotinine.

**Table 1:** Study Population for Cilia Length Measurements^1^.

Parameter	Paraffin-embedded endobronchial biopsy		Air-dried, unfixed brushed airway epithelium		Hydrated, unfixed brushed airway epithelium		Detached cilia air-dried, fixed, from brushed airway epithelium	
	Nonsmoker	Smoker	Nonsmoker	Smoker	Nonsmoker	Smoker	Nonsmoker	Smoker
N	15	13	18	21	6	10	13	14
Research subject label^2^	ns 1–15	s 1–13	ns 4, 7–11, 14–25	s 3, 4, 6, 12, 14–30	ns 7, 8, 26–29	s 3, 5, 27, 31–37	ns 2, 3, 5, 12, 13, 23–25, 29–33	s 7, 13, 20, 22, 23, 31, 33–36, 39–42
Sex (male/female)	14/1	10/3	12/6	14/7	4/2	6/4	11/2	10/4
Age (yr)	43 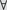 11	43 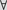 6	45 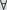 11	44 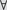 7	37 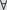 12	43 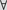 8	40 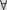 9	44 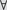 6
Race (Af/H/E/As/AH)^3^	8/3/4/0/0	10/2/1/0/0	5/2/10/1/0	12/3/6/0/0	4/0/1/0/1	6/3/1/0/0	9/1/3/0/0	9/3/2/0/0
**Smoking-related**								
Pack-yr	NA	29 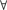 17	NA	32 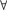 17	NA	34 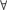 28	NA	33 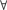 20
Cough and sputum score^4^	0.7 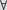 1.0	0.8 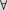 1.3	1.1 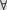 1.2	1.5 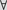 1.9	0.3 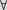 0.8	3.2 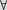 2.5	0.3 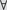 0.8	1.6 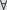 2.7
Urine nicotine (ng/ml)	0	845 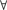 1122	0	708 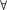 779	0	1681 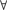 1660	0	1187 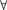 1644
Urine cotinine (ng/ml)	0	779 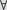 635	0	879 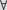 732	0	1276 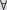 949	0	1056 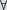 866
**Pulmonary function** 5								
FVC (% predicted)	114 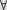 12	115 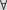 16	110 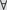 13	110 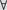 13	103 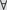 16	111 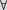 15	105 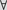 13	114 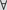 15
FEV1(% predicted)	115 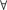 15	116 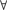 15	109 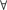 15	111 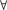 12	107 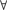 14	107 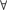 16	108 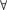 13	114 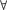 15
FEV1/FVC (% observed)	82 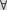 5	83 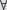 5	81 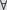 6	83 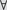 5	85 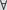 07	83 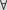 5	84 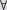 7	83 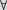 5
TLC (% predicted)	105 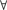 13	102 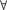 14	107 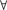 16	99 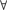 12	96 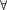 13	105 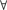 15	100 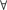 9	104 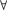 16
DLCO (% predicted)	101 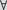 11	95 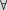 9	99 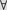 13	94 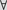 10	98 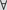 08	92 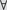 6	109 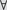 19	92 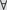 8

1 Biological samples from a total of 75 research subjects were used in four different analyses; in some cases, biological samples from a single research subject were used in more than one analysis (see footnote 2); where mean values are provided, the error indicates the standard deviation.

2 Research subject labels are not linked to any clinical research subject identifiers or clinical information; these labels have been generated for the express purpose of indicating where biological samples derived from a single research subject were used in multiple analyses.

3 Race is indicated as African (Af), Hispanic (H), European (E), Asian(As), African/Hispanic (AH).

4 Individual cough and sputum scores were based on a 0–5 Likert scale.

5 Pulmonary function: tests included forced vital capacity (FVC), forced expiratory volume in 1 sec (FEV1), total lung capacity (TLC), and diffusion capacity for carbon monoxide (DLCO).

### Sample Collection and Preparation

Large airway epithelium was obtained by fiberoptic bronchoscopy as previously described [3], [5]. After mild sedation was achieved with meperidine and midazolam, and routine anesthesia of the vocal cords and bronchial airways with topical lidocaine, a Pentax FB-15x fiberoptic bronchoscope (EB-1530T3, Orangeburg, NY) was positioned proximal to the opening of the desired lobar bronchus. Two methods were used to obtain airway epithelium, endobronchial biopsy and brushing.

Intact airway epithelial tissue was collected via endobronchial biopsy from segmental airway carinae of the right upper lobe (approximately 50%), right middle or lower lobes, left lower lobe, or lingula (combined approximately 50%). Biopsies were collected by advancing a pair of forceps through the working channel of the bronchoscope under direct visualization to the desired areas. The tissue was fixed in 4% paraformaldehyde, embedded in paraffin, and sectioned to a thickness of 5 :m. De-paraffinized sections were stained with hematoxylin and eosin.

To obtain airway epithelium by brushing, disposable brushes (2.0 mm, Ballard Medical, Draper, UT) were advanced through the working channel of the bronchoscope and used to brush and collect airway epithelial cells from 2^nd^ or 3^rd^ order bronchi from the right lower lobe by gently gliding the brush back and forth 10 times as previously described [3], [5], [41]. The collected cells were detached from the brush by flicking into 5 ml of bronchial epithelial basal cell medium (BEBM, Clonetics, Walkersville, MD). Three alternative methods were used to prepare the brushed airway epithelial cells.

First, cells were applied to microscope slides using centrifugal force. An aliquot of cells (2×10^4^ cells) was prepared by centrifugation (Cytospin 11, Shandon Instruments, Pittsburgh, PA), air-dried, fixed in 4% paraformaldehyde in PBS, and stained using Diff-Quik (Dade Behring, Newark, NJ).

Second, to assess the length of cilia that had become detached from the cells brushed from the epithelium, the cytospin, air-dried samples were stained with a mouse monoclonal anti-human ∃4 tubulin antibody (clone ONS1A6; 1/500 dilution; Biogenex, San Ramon, CA). Following incubation with the primary antibodies for 1 hr, 23°C in a humidified chamber, goat anti-mouse Cy3 conjugated AffiniPure F(ab′)_2_ (Jackson Immunoresearch, West Grove, PA) at 1/100 dilution was used as a secondary antibody.

Third, hydrated aliquots of cells were mounted on standard glass slides. Approximately 50 μl of cell suspension was placed on standard glass microscope slides. A 22×22 mm square coverslip with the edges coated with an inert silicon based grease (Dow Compound 111) was then placed on the droplet and the cell suspension dispersed evenly beneath the coverslip. Brightfield images of stained, air-dried ciliated cells, hydrated ciliated cells and paraffin- embedded biopsies were captured using a Nikon Microphot SA microscope using either a Plan Apo 40X N.A. 0.7 DIC objective lens, a Plan Apo 60X N.A. 1.4 DIC Oil objective lens, or a Plan Apo 100X N.A. 1.4 DIC Oil objective lens, and an Olympus DP70 color digital camera. Fluorescence microscopy was performed using an Olympus IX70 microscope with 100 watt mercury arc illumination using either a 60X N.A. 1.40 Plan Apo Oil objective lens or a UPlan Apo 100x N.A. 1.35 Oil objective with image capture by a Photometrix Quantix 57 cooled charge couple device (CCD; Universal Imaging, Downingtown, PA). Individual datasets derived from the overall subject cohort were chosen based on the availability of archived endobronchial biopsy samples, air-dried cytospins and samples of detached cilia; hydrated cilia were collected on a prospective basis.

### Sample Analysis

To evaluate cilia length in endobronchial biopsy samples, biopsies from 15 nonsmokers and 13 smokers were evaluated. Microscope slides with hematoxylin and eosin-stained biopsy tissue were evaluated by a blinded observer. Images were evaluated by digital image analysis using the MetaMorph image analysis package (Universal Imaging, Downingtown, PA). A stage micrometer with 10 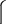
m gradations was used to calibrate images acquired from each objective lens. For each individual, a mean cilia length was calculated from at least 4 and as many as 13 individual measurements. The mean value for the group of individuals is presented as the average of the mean values for each individual 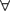 standard error of the mean.

To evaluate cilia length in air-dried cytospin samples, stained cytospin preparations from 18 nonsmokers and 21 smokers were evaluated. The cilia length of the bright field images was assessed in a blinded fashion as described above for biopsy samples. Multiple cells were evaluated in each cytospin preparation (range 19 to 60 measurements per individual). A mean cilia length was calculated for each individual, and the average of those means was taken as the mean for each group. The mean value for the group of individuals is presented as the average of the mean values for each individual 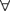 standard error of the mean.

To evaluate the length of detached cilia in cytospin preparations, staining for ∃4 tubulin was performed on cytospin samples from 13 nonsmokers and 14 smokers. Images were collected in a blinded manner and included cells carrying intact cilia, as indicated by their organization at the apical surface of the cell, as well as detached cilia with clear profiles that could be resolved from one end to the other. Only the latter were included in the analysis. Cilia lengths were determined for multiple detached cilia (range 5 to 103 measurements per individual). The mean value for the group of individuals is presented as the average of the mean values for each individual 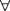 standard error of the mean.

To evaluate cilia length in hydrated airway cells, cell suspensions from 6 nonsmokers and 10 smokers were evaluated. Images of unstained, hydrated ciliated cells were collected using differential interference contrast optics in concert with the microscope and camera described above. The cilia lengths in the differential interference contrast images were assessed by digital tracing as described above. Multiple cells were evaluated in each cell suspension (range 17 to 46 cells per individual). Since samples had to be analyzed at the time that they were collected, blinding of the analysis was performed on the dataset of digital images before analysis of cilia length. A mean cilia length was calculated for each individual, and the average of those means was taken as the mean for each group. The mean value for the group of individuals is presented as the average of the mean values for each individual 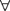 standard error of the mean.

### RNA and Microarray Processing

Large airway epithelium was obtained from a group of subjects (normal nonsmokers, n = 20, normal healthy smokers, n = 31, see Table S1) for RNA extraction and microarray analysis. Airway epithelial cells were obtained as described above. After flicking the cells into medium, an aliquot of 0.5 ml was used for differential cell count (typically 2×10^4^ cells per slide) and the remainder (4.5 ml) was processed immediately for RNA extraction. The total number of cells recovered by brushing was determined by counting on a hemocytometer. To quantify the percentage of epithelial and inflammatory cells and the proportions of basal, ciliated, secretory and undifferentiated cells recovered, cells were prepared by centrifugation (Cytospin 11, Shandon Instruments, Pittsburgh, PA) and stained with Diff-Quik (Baxter Healthcare, Miami, FL), and differential cell counts were performed. Only samples with <5% inflammatory cells were accepted for further analysis.

The HG-U133 Plus 2.0 array (Affymetrix, Santa Clara, CA), which includes probes for over 47,000 transcripts genome-wide, was used to evaluate gene expression. Total RNA was extracted using a modified version of the TRIzol method (InVitrogen, Carlsbad, CA), in which RNA is purified directly from the aqueous phase (RNeasy MinElute RNA purification kit, Qiagen, Valencia, CA). RNA samples were stored in RNA Secure (Ambion, Austin, TX) at 80°C. RNA integrity was determined by running an aliquot of each RNA sample on an Agilent Bioanalyzer (Agilent Technologies, Palo Alto, CA). The concentration was determined using a NanoDrop ND-1000 spectrophotometer (NanoDrop Technologies, Wilmington, DE). Double stranded cDNA was synthesized from 1 to 2 :g total RNA using the GeneChip One-Cycle cDNA Synthesis Kit, followed by cleanup with GeneChip Sample Cleanup Module, in vitro transcription (IVT) reaction using the GeneChip IVT Labeling Kit, and cleanup and quantification of the biotin-labeled cDNA yield by spectrophotometry. All kits were from Affymetrix (Santa Clara, CA). All HG-U133 Plus 2.0 microarrays were processed according to Affymetrix protocols, hardware and software, including being processed by the Affymetrix fluidics station 450 and hybridization oven 640 and scanned with an Affymetrix Gene Array Scanner 3000 7G. Overall microarray quality was verified by the following criteria: (1) RNA Integrity Number (RIN) >7.0; (2) 3′/5′ ratio for GAPDH <3; and (3) scaling factor <10.0.

### Web Deposition of Data

The raw data is all publically available at the Gene Expression Omnibus (GEO) site (http://www.ncbi.nlm.nih.gov/geo/), a high-throughput gene expression/molecular abundance data repository curated by the National Center for Bioinformatics (NCBI) site. The accession number for this data set is GSE16696.

### Microarray Data Analysis

Microarray data were processed using the MAS5 algorithm (Affymetrix Microarray Suite Version 5 software), which takes into account the perfect match and mismatch probes. MAS5-processed data were normalized using GeneSpring by setting measurements <0.01 to 0.01 and by normalizing per chip to the median expression value on the array and per gene to the median expression value across arrays. Expression was evaluated for 84 genes, represented by 188 probe sets, reported in the literature to be cilia-related. Genes that were significantly modified by smoking were selected according to the following criteria: (1) P call of “Present” in ∃20% of samples; (2) magnitude of fold change in average expression value for healthy smokers vs nonsmokers ∃1.5; and (3) p<0.01 using a t test with a Benjamini-Hochberg correction to limit the false positive detection rate.

### Statistics

To determine whether a significant difference in the distribution of mean cilia values was observed in nonsmoker and smoker populations (the data were assessed for normal distribution using a Shapiro-Wilk test), a standard two tailed t test was performed (StataCorp. 2007. Stata Statistical Software: Release 10. College Station, Tx: StataCorp) and p values <0.05 were taken as indicative of the fact that the mean values in the populations were significantly different. To test for correlations between clinical patient data and cilia length, a Pearson correlation analysis was employed (NCSS 97 statistics program, Kayesville, UT).

## Results

Subjects were recruited, consented and bronchoscoped for the study between December 12, 2004 and September 16, 2008. No serious adverse events were recorded during the study period. Individuals were followed for 7 days post-bronchoscopy for adverse events.

### Cilia Length in Fixed, Paraffin-Embedded Endobronchial Biopsy Specimens

**Figure 1:**
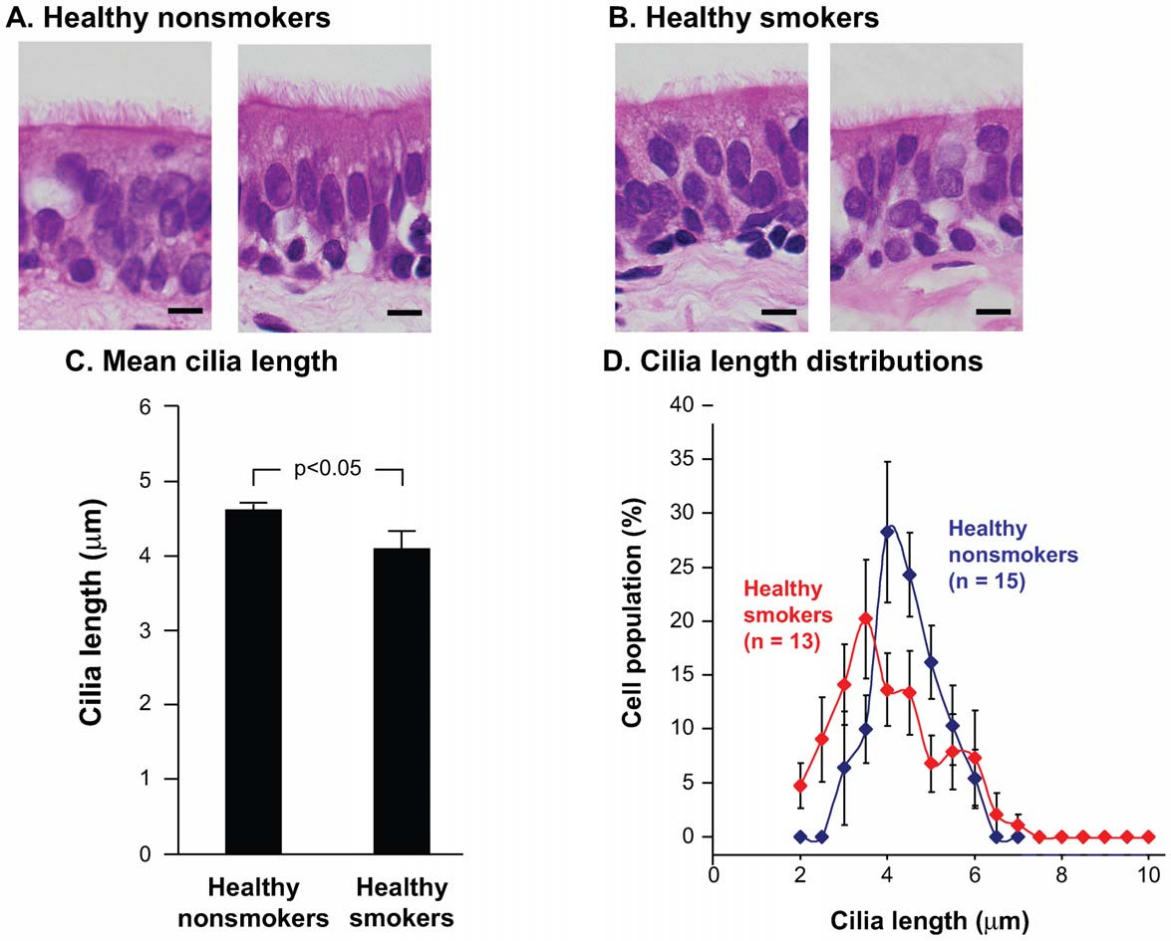
Cilia length in normal smokers and nonsmokers measured in fixed, paraffin-embedded endobronchial biopsies. Endobronchial biopsies were fixed in 4% paraformaldehyde, processed for paraffin embedding, sectioned at a thickness of 5 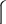
m, and stained with hematoxylin and eosin. Brightfield images were evaluated for cilia length. (A) Endobronchial biopsy morphology in normal nonsmokers. Bar  = 10 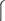
m. (B) Endobronchial biopsy morphology in normal smokers. Bar  = 10 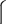
m. (C) Mean cilia length within the study populations. Shown are the mean 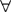 standard error of cilia lengths for nonsmokers (n = 15) and smokers (n = 13). Between 4 and 13 individual measurements were made per study individual (median  = 7). (D) Distribution of cilia lengths in airway epithelial cells from normal nonsmokers and normal smokers. Distributions were constructed by creating histograms from 0.5 micron bins and then equally weighting each study individual.

Assessment of cilia length in endobronchial biopsies obtained demonstrated that the cilia were shorter in smokers compared to nonsmokers (Figure 1A and 1B). Nonsmokers had an average cilia length of 4.7 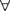 0.1 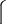
m while smokers had an average length of 4.0 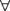 0.3 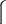
m (p<0.05), reflecting a 15% decrease in cilia length for the smokers (Figure 1C). When the distributions of cilia lengths were averaged across all 15 nonsmokers and 13 smokers, weighting each study individual equally, the distribution of cilia lengths in smokers was shifted toward shorter cilia (Figure 1D). Remarkably, 23% of smokers had a mean value for cilia length that fell below the lowest mean cilia length observed among 15 non-smoking individuals (Figure S1).

### Cilia Length in Air-Dried, Fixed-Airway Epithelial Cells

**Figure 2:**
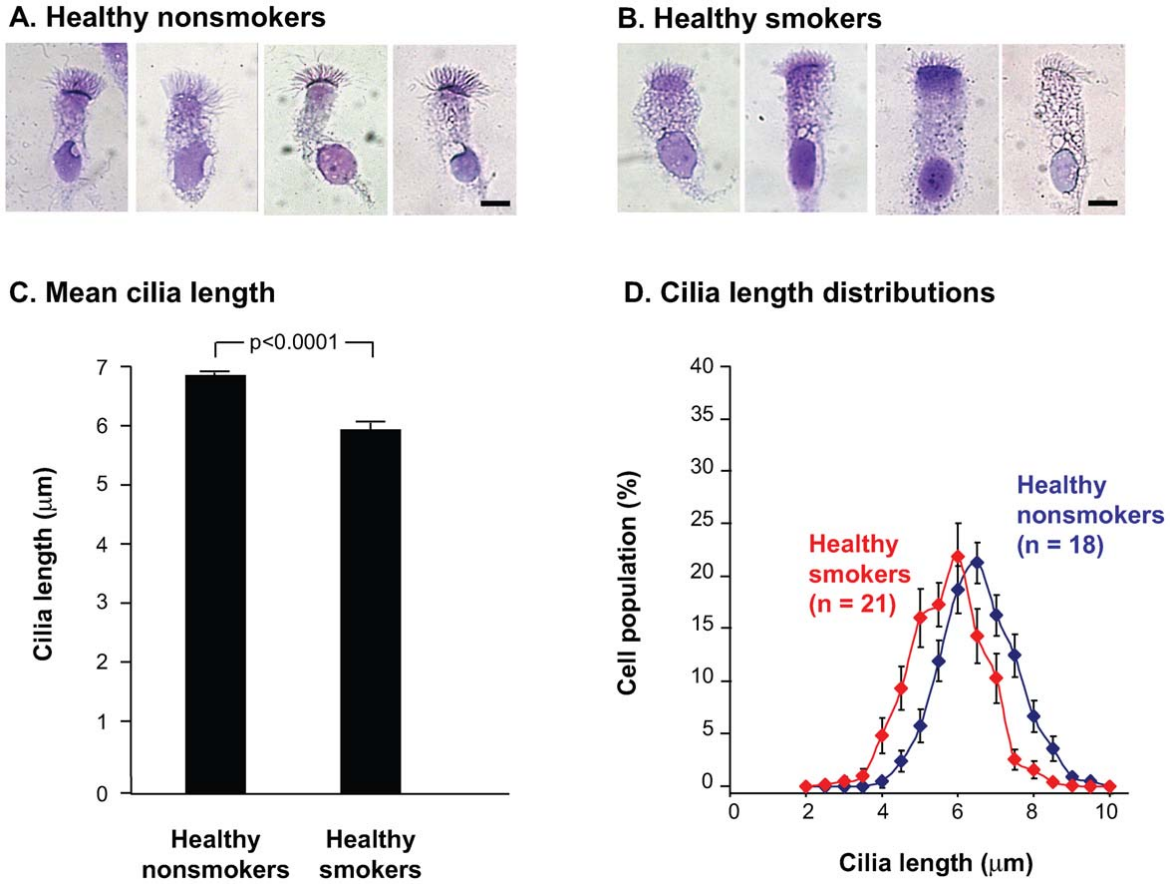
Cilia length in normal smokers and nonsmokers measured in air-dried, un-fixed samples. Suspensions of airway epithelial cells were applied to glass slides using a cytocentrifuge, air-dried, and stained with Diff-Quik. (A) Ciliated airway epithelial cell morphology in normal nonsmokers. Bar  = 10 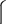
m. (B) Ciliated airway epithelial cell morphology in normal smokers. Bar  = 10 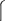
m. (C) Quantitative assessment of cilia length of normal nonsmokers and normal smokers. Shown are the mean 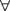; standard error of cilia lengths for nonsmokers (n = 18) and smokers (n = 21). Between 19 and 60 individual measurements were made per study individual (median  = 27). (D) Distribution of cilia lengths in airway epithelial cells from normal nonsmokers and normal smokers. Distributions were constructed by creating histograms from 0.5 micron bins and then equally weighting each study individual.

Ciliated cells brushed from the airways of normal nonsmokers and normal smokers were evaluated following cytocentrifugation, air-drying, fixation, and staining. Upon inspection, the epithelium from the smokers exhibited apparently shortened cilia compared to the nonsmokers (Figure 2A and 2B). Quantitative analysis of the cilia showed that nonsmokers had an average cilia length of 6.8 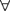 0.1 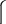
m while smokers had an average length of 5.9 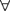 0.1 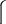
m (p<0.0001; Figure 2C). The data reflected a 13% decrease in average cilia length in smokers compared to nonsmokers. The distributions of cilia lengths weighted equally based on the 18 nonsmoker and 21 smoker individuals evaluated showed a shift toward shorter cilia (Figure 2D). The measured lengths of air-dried, fixed cilia for both the nonsmokers and smokers were significantly longer than cilia lengths measured in paraffin-embedded biopsy samples, likely reflecting a shrinkage artifact induced by the paraffin embedding procedure [42], but the proportional difference between smokers and nonsmokers was similar. As observed with the paraffin-embedded sections, a large degree of individual variability was observed among smokers with nearly 50% of smokers showing a lower mean cilia length than the lowest mean cilia length observed among 18 normal nonsmokers (Figure S1). To determine whether there was a correlation between smoking history (pack-yr) and cilia length, a Pearson correlation analysis was performed resulting in an *r* value of 0.16 and a p value of 0.48, indicating that no significant correlation existed.

In order to assess the variability of cilia length in individual cells we measured multiple cilia on 5 randomly selected cells from 5 nonsmokers and 5 smokers randomly selected from our cohort. The distribution of cilia length among healthy nonsmokers and healthy smokers is demonstrated in Figure S2 where the mean length of cilia for an individual cell is shown for both populations. The variability was similar in both the healthy nonsmokers and healthy smokers where the mean standard deviation of cilia length in healthy nonsmokers was 0.8 µm and in healthy smokers 0.7 µm.

### Length of Detached Cilia in Air-Dried, Fixed-Airway Epithelial Cells

**Figure 3:**
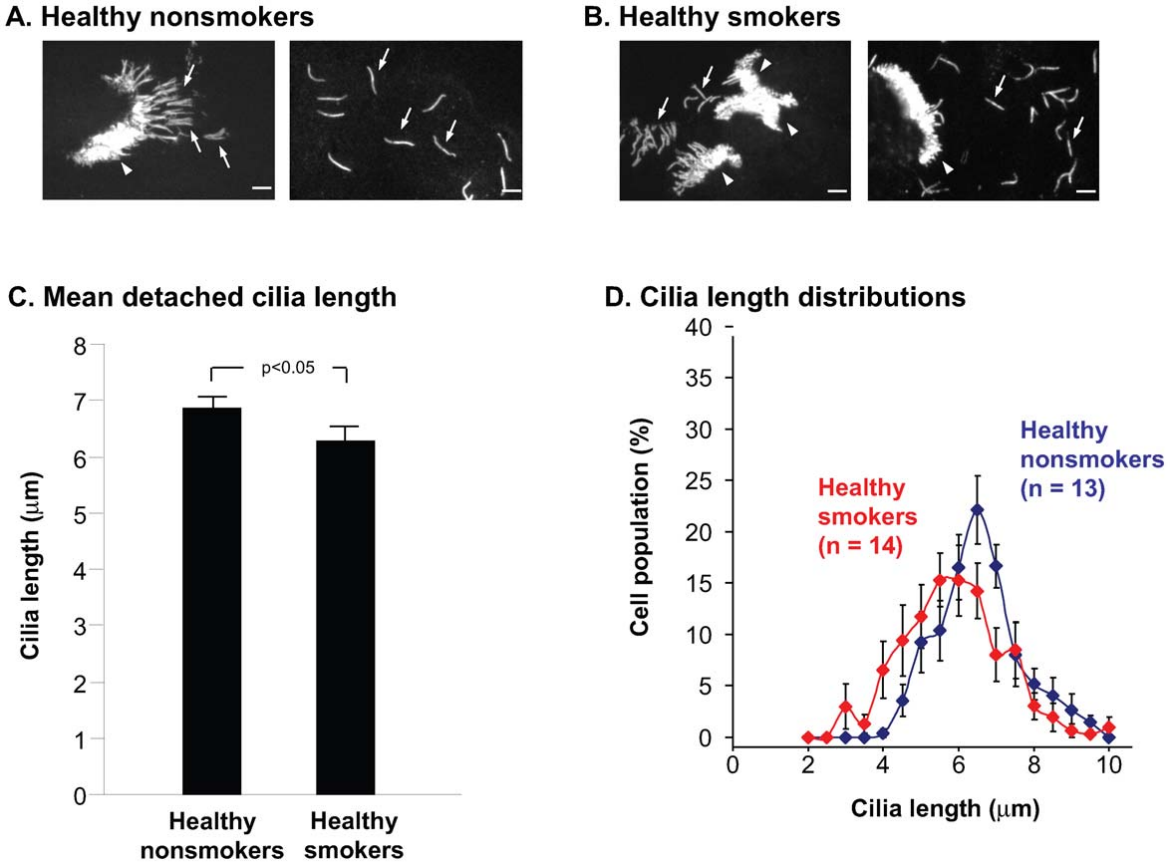
Cilia length in detached cilia from airway epithelial cells of normal smokers and nonsmokers. Suspensions of airway epithelial cells were applied to glass slides using a cytocentrifuge, air-dried, fixed in 4% paraformaldehyde, and stained with an antibody against ∃4-tubulin. Fields include cilia that were detached from cells (arrows) as well as cilia that remain attached to the apical surface of cells (arrowheads). Only detached cilia that were visible from end-to-end were included in the analysis. (A) Detached cilia in airway epithelial cells from normal nonsmokers. Bar  = 5 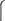
m. (B) Detached cilia in airway epithelial cells from normal smokers. Bar  = 5 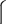
m. (C) Quantitative assessment of cilia length in detached cilia from normal nonsmokers and normal smokers. Shown are the mean 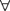 standard error of cilia lengths for nonsmokers (n = 13) and smokers (n = 14). Between 5 and 103 individual measurements were made per study individual (median  = 54). (D) Distribution of cilia lengths in airway epithelial cells from normal nonsmokers and normal smokers. Distributions were constructed by creating histograms from 0.5 micron bins and then equally weighting each study individual.

Measurements of cilia length in DiffQuik-stained cells in cytospin preparations represented an assessment of the average length of the tuft of cilia at the apical end of each epithelial cell. To confirm that the difference in cilia length was a property of individual cilia, detached cilia were identified on cytospin preparations of air-dried brushed samples from normal nonsmokers and normal smokers and evaluated after indirect immunofluorescence staining of ∃4 tubulin. Both intact and detached cilia were evident at high magnification (Figure 3A and 3B). Quantitative analysis of the detached cilia showed that nonsmokers had an average cilia length of 6.9 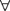 0.2 µm while smokers had an average length of 6.3 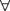 0.3 µm (p<0.05; Figure 3C). The data reflected a 9% decrease in average cilia length in smokers compared to nonsmokers. The distributions of cilia lengths weighted equally based on the 13 nonsmoker and 14 smoker individuals evaluated showed a shift toward shorter cilia (Figure 3D). When comparing the individual means for the two populations, it was noted that 29% of smokers displayed mean cilia lengths shorter than all of the 13 nonsmokers in the study (Figure S1).

### Cilia Length in Hydrated, Unfixed Ciliated Airway Epithelial Cells

**Figure 4:**
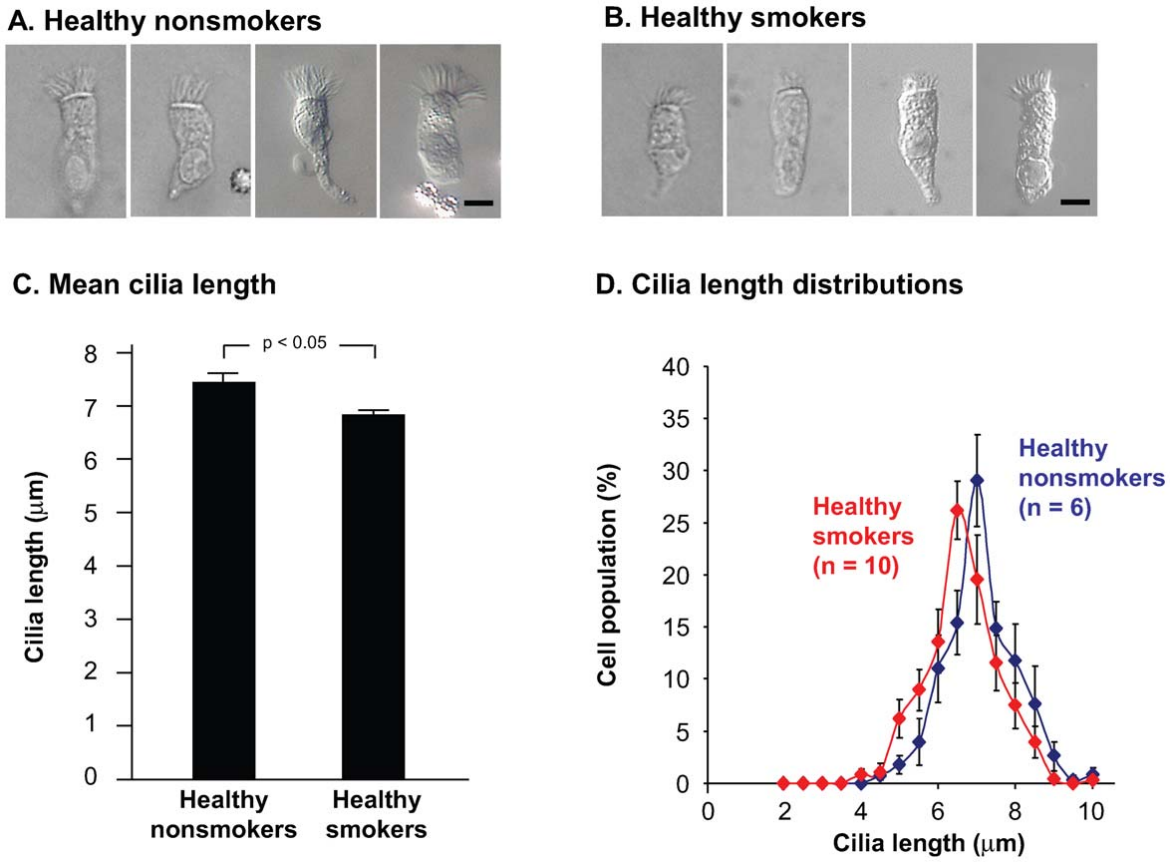
Cilia length in normal smokers and nonsmokers measured in unfixed, hydrated cells. Freshly isolated suspensions of airway epithelial cells were added to coverslip chambers and imaged using differential interference contrast microscopy. (A) Ciliated airway epithelial cell morphology in normal nonsmokers. Bar  = 10 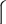
m. (B) Ciliated airway epithelial cell morphology in normal smokers. Bar  = 10 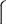
m. (C) Mean cilia length within the study populations. Shown are the mean 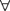 standard error of cilia lengths for nonsmokers (n = 6) and smokers (n = 10). Between 17 and 46 individual measurements were made per study individual (median  = 24.5). (D) Distribution of cilia lengths in airway epithelial cells from normal nonsmokers and normal smokers. Distributions were constructed by creating histograms from 0.5 micron bins and then equally weighting each study individual.

Cilia lengths for both smokers and nonsmokers quantified in air-dried fixed airway epithelium recovered by brushing (Figure 2 and Figure 3) were longer than those observed in paraffin embedded sections (Figure 1) and were comparable to cilia lengths reported by other investigators [36]. As noted above, the difference compared to paraffin embedded sections likely reflected the absence of a dehydration artifact during paraffin embedding. To test whether air drying introduced other cilia length artifacts, cilia length was also evaluated in hydrated, unfixed cells. Morphological observations and cilia length measurements of cytospin preparations of hydrated, unfixed ciliated cells from normal nonsmokers and normal smokers were consistent with those of air-dried, fixed samples (Figure 4A and 4B). Cilia were significantly shorter in smokers compared to nonsmokers with an average length of 6.8 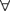 0.1 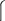
m compared to 7.3 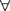 0.2 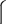
m (p<0.05) with an average decrease in the population cilia length of 7% (Figure 4C). The distribution of cilia lengths equally weighted across samples from 6 nonsmokers and 10 smokers showed a shift toward the shorter cilia (Figure 4D), consistent with observations above using different sample preparation methods. When comparing cilia lengths in air-dried *vs* hydrated samples in the nonsmokers or smokers, it was clear that measurement of hydrated cells yielded a cilia length that was approximately 10% longer than observed in air-dried samples prepared either by DiffQuik stain or by fluorescence stain for ∃4 tubulin. Based on this analysis, cilia lengths measured on air-dried, fixed, stained cytological preparations were taken to be accurate representations of cilia length *in situ*.

Consistent with the biopsy and cytospin preparations described above, an analysis of the individual mean cilia lengths for the individuals in the study of hydrated cells revealed that 36% of smokers had a mean cilia length that was shorter than all of the 6 nonsmoker samples examined (Figure S1). To better understand the variability of cilia length exhibited by cells from a single donor, the distributions of cilia lengths were plotted separately for each individual who contributed cells to the hydrated cell analysis (Figure S3). These data showed that 4 out of the 10 smokers had cilia length distributions in which at least 25% of cells carried cilia shorter than 6 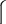
m whereas none of the 6 nonsmokers analyzed had 25% of cells with cilia lengths shorter than 6 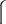
m (Figure S3).

### Theoretical Reduction in the Population of Cilia Contributing to Mucociliary Clearance

**Figure 5:**
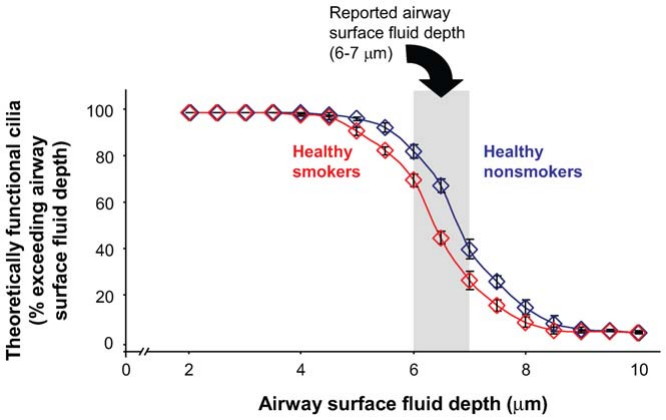
Theoretical effect of smoking on ciliary contribution to mucociliary clearance. The models of cilia/mucus interaction propose that the tip of the cilia must pass through the periciliary fluid to contact the mucus in order to generate mucus movement [15], [17], [39], [40]. Using the data generated in the study of hydrated, unfixed airway epithelial cells, the graph shows the proportion of cilia that would extend through the airway surface fluid (y-axis, “effective cilia”) at a variety of hypothetical airway surface fluid depths (x-axis).The range of reported normal depths of airway surface fluid is shown in gray. Proportions of effective cilia are shown for nonsmokers (blue) and smokers (red).

All four methods used to assess cilia length showed a decrease in cilia length in the airway epithelial cells of healthy smokers compared with nonsmokers. Models for mucociliary clearance call for cilia to extend through the airway surface fluid to contact the mucus in order to generate a propulsive force [15], [17], [39], [40]. Using data from the study of hydrated cells, the percentage of cilia capable of reaching mucus at different heights can be represented (Figure 5). In this model, due to the shift toward shorter cilia, smokers consistently have a lower percentage of cells with cilia that could reach through the airway surface fluid to contact the mucus at a given airway surface fluid depth. The actual depth of the airway surface fluid is reported to be 6 to 7 μm [43]–[47]. In this range, the shorter cilia in smokers would result in a reduction of effective cilia of approximately 18 to 38%, consistent with the data that smokers have a decreased mucociliary clearance compared to nonsmokers [1], [7], [8], [18]–[24].

### Effect of Smoking on Cilia-Related Gene Expression in the Large Airway Epithelium

Using the criteria of P call of “Present” in ∃20% of samples, magnitude of fold-change in healthy smokers *vs* nonsmokers ∃1.5, and p<0.01 using a t test and a Benjamini-Hochberg correction to limit the false positive rate, 9 cilia-related probe sets were identified as having expression levels significantly responsive to smoking in the large airway epithelium (see Table S2). Of these, 6 genes were represented. All differentially expressed genes were downregulated in smokers. Two genes involved in the function of the cilia basal body, outer dense fiber of sperm tails 2 (ODF2) and ezrin (EZR), were significantly downregulated in smokers as compared to nonsmokers (p<0.005 for both). Three axonemal dyneins were also significantly downregulated in smokers: dynein, axonemal, heavy chain 5 (DNAH5); dynein, axonemal, heavy chain 10 (DNAH10); and dynein, axonemal, heavy chain 11 (DNAH11) (p<0.003 for each). Finally a cytoplasmic dynein, dynein cytoplasmic 2 heavy chain 1 (DYNC2H1), which is the retrograde motor of the cilia, was significantly downregulated in smokers (p<0.001).

## Discussion

Healthy smokers with normal lung function and normal chest X-rays are at significant risk for respiratory tract infections, chronic obstructive lung disease and bronchogenic carcinoma [9]–[13]. Fundamental to these risks are the observations that cigarette smoking is associated with a decrease in mucociliary clearance, a process driven by ciliated airway epithelial cells functioning in a coordinated fashion to move airway surface fluid and mucus in a cephalad fashion, thus continually cleansing the respiratory surface of inhaled particulates [1], [7], [8]. Prior reports have attributed the smoking-related decrease in mucociliary clearance to a decrease in numbers of ciliated cells, changes in cilia structure and/or beat frequency [18]–[34], [36]. While these mechanisms likely contribute to the smoking-induced dysfunction in mucociliary clearance, the present study documents a new concept to help explain decreased mucociliary clearance, that smoking is associated with an average shortening of airway epithelial cilia. Independent of the methodology used to assess airway epithelial cilia length in normal smokers compared to nonsmokers, the results consistently demonstrate that, on average, cilia of normal smokers are 10% shorter than those of normal nonsmokers. Based on models of mucociliary clearance, this reduction in cilia length should have a significant influence on mucociliary clearance, and thus is likely to have a significant role in the risk for developing smoking-induced lung disease.

Perhaps of greater significance is the potential implication for individual smokers. As noted in each method of analysis, a significant population of smokers (23 to 50%) exhibited mean cilia lengths that were shorter than the minimum mean cilia length observed for nonsmokers using the same analytical technique. To understand the significance of these data at the level of individual cells, plots of the distribution of cilia lengths observed in hydrated, unfixed cells were plotted for each individual in the study, and the results confirmed that 4 out of 10 smokers had a large fraction of cells (>25%) with cilia shorter than 6 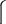
m, a length that theoretically might not contribute to mucus flow. The basis for the individual variation among smokers did not correlate with pack-yr history of smokers, so other factors possibly including genetic predisposition may be involved.

### Prior Studies of Airway Epithelial Cilia Length

The potential influence of shortened cilia on mucociliary clearance has received little attention. Chang [36] demonstrated a decrease in cilia length in smokers compared to nonsmokers, but in this study, autopsy material was obtained from both healthy and diseased lungs, making it difficult to isolate the effects of smoking and other sequalae of smoking. Serafini and Michaelson [37] examined cilia lengths at different levels of the airway, concluding that cilia length decreased with increasing order of bronchioles. In this study, relatively short cilia were reported in a single patient with COPD, but the smoking status of that patient was not reported. Nagai and Thurlbeck [38] noted the presence of short cilia in the airway of patients with emphysema, but smoking status was not included in this qualitative study. Decreased cilia length has also been reported in humans exposed to sulfur dioxide, ozone, and following viral infection [48]–[51]. Studies in experimental animals with acute and chronic ozone exposure show that the airway responds to acute exposure by sloughing airway epithelium, but eventually compensates for the initial airway toxicity by rebuilding an intact, apparently healthy airway except that cilia remain shortened [52]–[54]. Viral infection and cigarette smoke have been shown to decrease cilia length in rodents, but these studies as well as the ozone studies were not quantitative with respect to cilia length [55], [56].

### Regulation of Cilia Length

The results of our quantitative analysis of airway epithelial cilia length demonstrate that smokers have shorter cilia, but do not identify the biologic mechanisms by which cigarette smoke results in shorter cilia. Cilia, like other organelles, are constantly broken down and regenerated, and it is likely that cigarette smoke, with its many toxic components, affects multiple biologic processes involved in cilia growth. In our study using genome-wide expression analysis of human large airway epithelium we found 6 cilia-related genes that were significantly down-regulated in healthy smokers compared to healthy nonsmokers. Three of these were axonemal dynein genes. Dyneins are involved in ciliary movement and mutations in DNAH 5 and 11 have been reported in primary cilia dyskinesia [57], [58]. Ezrin and ODF2 (also known as cenexin) are proteins associated with basal body development. Ezrin acts as an intermediate between the plasma membrane and the actin cytoskeleton. In ezrin knockout mice, loss of ezrin has been reported to be associated with substantial reduction in length of the apical micro-villi of the retinal epithelium [59], but its role in respiratory epithelium is not yet known. Odf2 was originally identified as a component of the sperm flagellae but subsequent studies have shown it to be a general scaffold protein that is specifically localized at the distal/subdistal appendages of the centrosome which are required for centriolar maturation. Ishikawa et al reported that Odf2 knockout mice lack these distal appendages during centriolar formation which results in a failure to develop primary cilia [60]. Finally DYNC2H1 was also shown to be down-regulated in healthy smokers. DYNC2H1 is a cytoplasmic dynein involved in the retrograde transport of axonemal proteins from the distal tip of the cilium to the basal body. This is an integral part of cilia homeostasis and possibly preservation of cilia length through the dynamic process of intraflagellar transport (IFT). Cultured chondrocytes from affected individuals with mutations in DYNC2H1 demonstrate shortened and bulbous cilia when compared to controls [61]. Other genes that have been previously identified to be associated with the control of cilia and flagellar length in lower eukaryotes such as DYNC2LI1 in *Tetrahymena thermophilia* and IFT88 in *Chlamydomonas* were not affected by cigarette smoking in our study [62], [63].

### Implications of Reduction of Cilia Length on Mucociliary Clearance

Theoretical models of cilia beat and force generation in the airway epithelium predict that the ability of cilia to propel mucus in a unidirectional manner results from a preferential interaction of cilia tips with mucus on the forward stroke while the cilia beat incorporates an oblique bend on the reverse stroke, thus effectively shortening the cilia which passes through the airway surface fluid without contacting the mucus [15], [17], [39], [40]. These models predict that only the distal 10% of the cilia length makes contact with the mucus on the forward stroke. Our observations demonstrate decrease in mean cilia length in normal smokers, suggesting that the short cilia in the airway epithelium of smokers might exhibit reduced interaction with mucus on the forward stroke. As a result, the short cilia on the airway surface of smokers is likely to contribute to the reduction in mucociliary clearance rates in smokers. In addition to the reduced ability to interact with mucus on the forward stroke, a overall reduction in cilia length would have a net effect on the generation of force in the mucus layer. A decreased cilia length would translate into a decreased motive force since radius and torque are proportional and linearly related. Thus, a 10% decrease in the radius of the forward stroke would be expected to decrease the torque of a cilium by 10%. It is also interesting to speculate whether reduced dynein heavy chain expression contributes to reduced ciliary beat frequency which is a known effect of cigarette smoking [18], [19]. Individuals with shorter cilia would potentially also be at further increased risk of poor mucociliary clearance due to the mechanical disadvantage conferred by both defects. It is likely that a combination of factors, including a reduction in cilia length, could contribute to a reduction in airway mucus clearance. In our dataset we compared ciliary length to cough and sputum scores but found no correlation. This is not surprising since the likely impact in healthy smokers of shorter cilia would be difficult to quantify with a relatively crude qualitative instrument such as the cough and sputum score. Further studies using tests of mucocillary clearance would be needed to measure the effects of ciliary length on mucociliary clearance and mucus velocity.

One potential compensation for cilia shortening in smokers would be a concomitant reduction in airway surface fluid height that would re-establish the relationship between cilia and the mucus such that 10% of the cilia length would enter the mucus on the forward stroke regardless of the cilia length. Such an interdependence of cilia height and airway surface fluid height based on adhesion of airway surface fluid to cilia has been proposed [15]. However, recent observations demonstrate that airway surface fluid height is a function of salt concentration rather than cilia height [47]. In addition, freeze substitution studies indicate that the airway surface fluid height is constant regardless of the extension or flexion of cilia during various stages of the cilia beat cycle [43]. If airway surface fluid height is constant and cilia length is reduced in smokers, then shorter cilia will contribute to reduced mucociliary clearance.

Airway surface fluid height has been proposed to range from 6 to 7 µm [43]–[47]. Based on theoretical models of the effect of smoking on mucociliary clearance, the percentage of functional cilia would decrease approximately 18 to 38% in smokers based on cilia height alone. If the cilia must reach the mucus layer, then this significant decrease in the percentage of effective cilia in the 6 to 7 ?m range would result in depressed mucociliary clearance as there would be fewer cilia to generate a force to propel the layer forward. If the smoking-related reduction in airway epithelial cilia length does impact mucociliary clearance as predicted by these models, then the reduction in cilia length likely plays a significant role in the pathogenesis of the smoking-induced lung disorders. A recent revised model of the relationship between mucus and airway surface fluid suggests that mucus forms a continuum from gel to sol as it approaches the apical surface of airway epithelial cells [45], [64], [65]. While this model may not envision a threshold length for cilia to be effective, the impact of a general decrease in cilia length would be likely to have similar consequences due to decreased interaction of cilia with the mucus gel phase.

Since it is ethically difficult to measure cilia length before and after initiation of smoking in humans we sought evidence in the literature for causality and found a recently published report from Tamashiro et al [66] demonstrating in an *in vitro* air-liquid interface model of cultured mouse nasal epithelium that exposure to cigarette smoke condensate (the particulate phase) and cigarette smoke extract (the volatile phase) resulted in shortened cilia when compared to controls and that the relationship was dose dependent [66].

Finally, we hope that this study gives us new insights into acquired ciliopathies due to the effects of cigarette smoke and improves our understanding of the biologic processes underlying airway epithelial cilia growth, and the mechanisms of how cigarette smoke mediates a reduction in cilia length.

## Supporting Information

Figure S1 Plot of mean cilia lengths from each individual in the study. (A) Data from paraffin-embedded biopsies corresponding to Figure 1. (B) Data from air-dried, fixed cells corresponding to Figure 2. (C) Data from detached cilia corresponding to Figure 3. (D) Data from hydrated, unfixed cells corresponding to Figure 4. Note that a high percentage of individual smokers (23%, 50%, 29%, and 36%, respectively) exhibited a mean cilia length that was less than the minimum mean cilia length observed in all nonsmokers for the same analysis (dotted lines). These data suggest that individual smokers may exhibit a greater risk for shortened cilia.
(0.02 MB PDF)

Figure S2 Plot of cilia length in individual cells in 5 randomly selected smokers and 5 nonsmokers. Each individual had 10 cilia length measurements per cell, 5 cells per individual. Results are expressed as the mean cilia length per cell with the standard deviation. Each individual is labeled by a specific colored icon; healthy nonsmokers triangles, healthy smokers diamonds.
(0.03 MB PDF)

Figure S3 Plot of cilia length distributions for each individual in the hydrated, unfixed cell study. To better understand the variability of cilia length from cell-to-cell within an individual, the distribution of cilia lengths within each individual in the hydrated cell study were plotted (data correspond to Figure 4). Raw cilia length data were collected in 0.5 µm bins (x-axis) and the % of the population in each bin was plotted on the y-axis. Graphs have been displaced on the y-axis to clarify the individual distributions. The theoretical length of cilia needed to extend through the airway epithelial lining fluid is shaded in gray. Of interest, in 4 out of 10 smokers, >25% of individual cells were observed to have cilia lengths that fell below the theoretical length needed to contribute to mucus movement. These data, as well as the data in Figure S1, suggest that individual smokers may exhibit a greater risk for shortened cilia.
(0.02 MB PDF)

Table S1 Study Population for Large Airway Epithelial Gene Expression.
(0.01 MB PDF)

Table S2 Cilia-Related Gene Expression in Airway Epithelium.
(0.04 MB PDF)
