# Supplementary figures and images for: Smoking Is Associated with Shortened Airway Cilia
Source: PLoS One. 2009 Dec 16;4(12):e8157. doi: 10.1371/journal.pone.0008157 (PMC2790614; doi:10.1371/journal.pone.0008157)

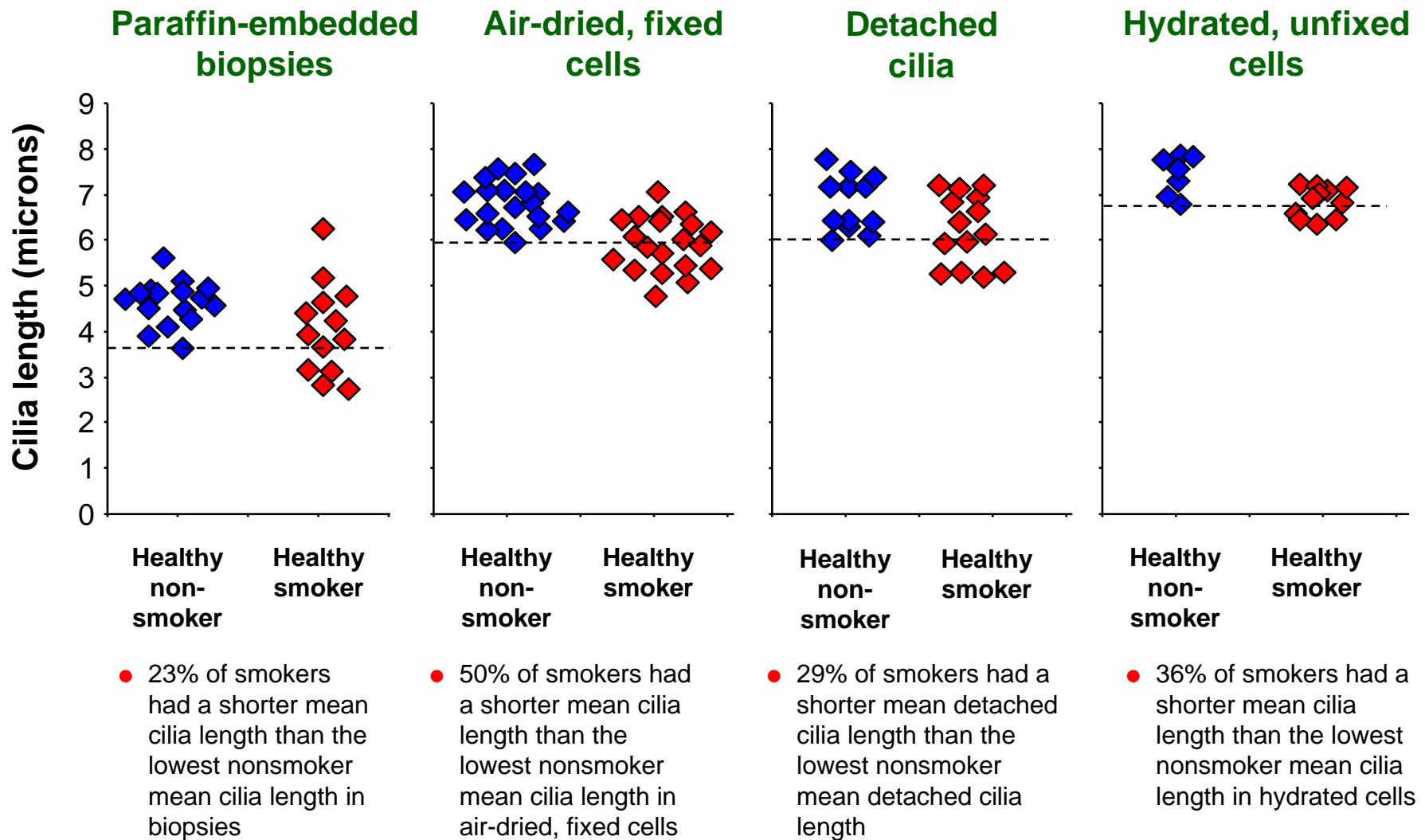

Supplement: Figure S1 — Plot of mean cilia lengths from each individual in the study. (A) Data from paraffin-embedded biopsies corresponding to Figure 1. (B) Data from air-dried, fixed cells corresponding to Figure 2. (C) Data from detached cilia corresponding to Figure 3. (D) Data from hydrated, unfixed cells corresponding to Figure 4. Note that a high percentage of individual smokers (23%, 50%, 29%, and 36%, respectively) exhibited a mean cilia length that was less than the minimum mean cilia length observed in all nonsmokers for the same analysis (dotted lines). These data suggest that individual smokers may exhibit a greater risk for shortened cilia. (0.02 MB PDF) [file pone.0008157.s001.pdf]

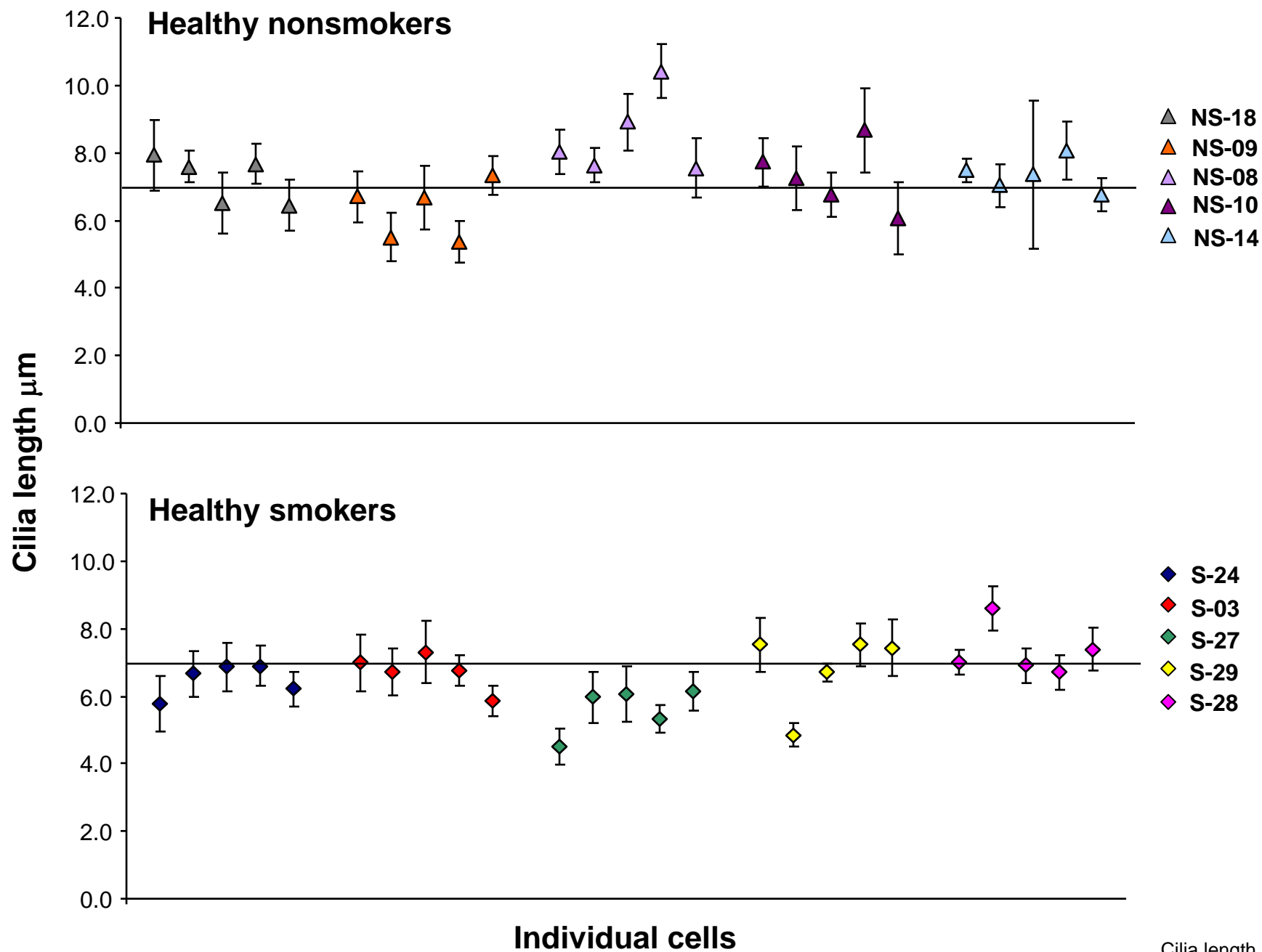

Supplement: Figure S2 — Plot of cilia length in individual cells in 5 randomly selected smokers and 5 nonsmokers. Each individual had 10 cilia length measurements per cell, 5 cells per individual. Results are expressed as the mean cilia length per cell with the standard deviation. Each individual is labeled by a specific colored icon; healthy nonsmokers triangles, healthy smokers diamonds. (0.03 MB PDF) [file pone.0008157.s002.pdf]

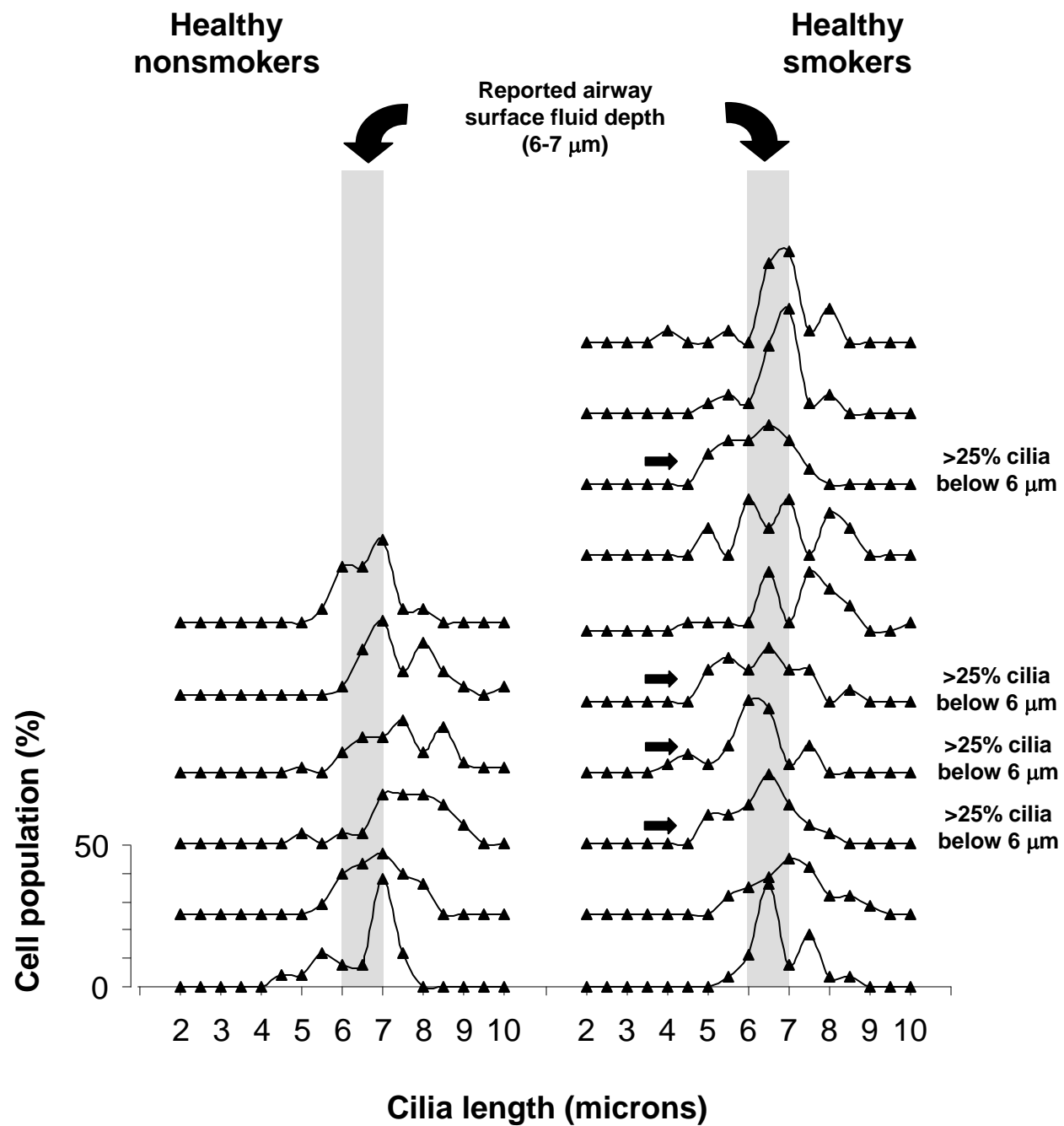

Supplement: Figure S3 — Plot of cilia length distributions for each individual in the hydrated, unfixed cell study. To better understand the variability of cilia length from cell-to-cell within an individual, the distribution of cilia lengths within each individual in the hydrated cell study were plotted (data correspond to Figure 4). Raw cilia length data were collected in 0.5 µm bins (x-axis) and the % of the population in each bin was plotted on the y-axis. Graphs have been displaced on the y-axis to clarify the individual distributions. The theoretical length of cilia needed to extend through the airway epithelial lining fluid is shaded in gray. Of interest, in 4 out of 10 smokers, >25% of individual cells were observed to have cilia lengths that fell below the theoretical length needed to contribute to mucus movement. These data, as well as the data in Figure S1, suggest that individual smokers may exhibit a greater risk for shortened cilia. (0.02 MB PDF) [file pone.0008157.s003.pdf]
