# Supplementary material for: Smoking Is Associated with Shortened Airway Cilia
Source: PLoS One. 2009 Dec 16;4(12):e8157. doi: 10.1371/journal.pone.0008157 (PMC2790614; doi:10.1371/journal.pone.0008157)
Supplement: Table S1 — Study Population for Large Airway Epithelial Gene Expression. (0.01 MB PDF) [file pone.0008157.s004.pdf]

Supplemental Table I. Study Population for Large Airway Epithelial Gene Expression[1]

Parameter	Large airway gene expression	
	Nonsmoker	Smoker
n	20	31
Sex (male/female)	14/6	21/10
Age (yr)	40 ± 8	44 ± 7
Race (Af/H/E/As/AH)[2]	8/3/7/1/1	20/5/6/0/0
**Smoking-related**		
Pack-yr	NA	28 ± 18
Urine nicotine (ng/ml)	0	1059 ± 1088
Urine cotinine (ng/ml)	0	1099 ± 1091
**Pulmonary function**[3]		
FVC (% predicted)	107 ± 13	112 ± 11
FEV1(% predicted)	103 ± 28	110 ± 12
FEV1/FVC (% observed)	82 ± 5	82 ± 7
TLC (% predicted)	100 ± 14	103 ± 12
DLCO (% predicted)	101 ± 16	96 ± 12

1 Biological samples from a total of 51 research subjects were used to assess large airway gene expression; where mean values are provided, the error indicates the standard deviation.

2 Race is indicated as African (Af), Hispanic (H), European (E), Asian(As), African/Hispanic (AH).

3 Pulmonary function: tests included forced vital capacity (FVC), forced expiratory volume in 1 sec (FEV1), total lung capacity (TLC), and diffusion capacity for carbon monoxide (DLCO).
