# Supplementary material for: Smoking Is Associated with Shortened Airway Cilia
Source: PLoS One. 2009 Dec 16;4(12):e8157. doi: 10.1371/journal.pone.0008157 (PMC2790614; doi:10.1371/journal.pone.0008157)
Supplement: Table S2 — Cilia-Related Gene Expression in Airway Epithelium. (0.04 MB PDF) [file pone.0008157.s005.pdf]

Supplemental Table II: Cilia-related Gene Expression in Airway Epithelium[1]

Gene symbol	Probe set ID	Gene title	P call (%)	Fold-change[2]	p value[3]	Reference
AKAP14	237281_at	A kinase (PRKA) anchor protein 14	100	-1.17	$1.18x10^{-01}$	[1]
AKAP14	237282_s_at	A kinase (PRKA) anchor protein 14	100	-1.43	$1.35x10^{-02}$	[1]
AKAP14	242698_at	A kinase (PRKA) anchor protein 14	57	-1.59	$4.75x10^{-02}$	[1]
ALMS1	214220_s_at	Alstrom syndrome 1	100	-1.18	$4.16x10^{-02}$	[2]
ALMS1	214221_at	Alstrom syndrome 1	100	-1.08	$7.02x10^{-01}$	[2]
ALMS1	214707__at	Alstrom syndrome 1	100	1.15	$4.86x10^{-01}$	[2]
BBS1	218471_s_at	Bardet-Biedl syndrome 1	100	-1.08	$5.42x10^{-01}$	[3,4]
BBS1	222643_s_at	Bardet-Biedl syndrome 1	75	-1.34	$6.62x10^{-02}$	[3,4]
BBS1	229142_s_at	CDNA FLJ52492 complete cds, highly similar to Bardet-Biedl syndrome 1 protein	4	-1.15	$6.60x10^{-01}$	[3,4]
BBS2	223227_at	Bardet-Biedl syndrome 2	100	-1.x10	$2.x10x10^{-01}$	[5]
BBS4	212744_at	Bardet-Biedl syndrome 4	100	-1.x10	$3.61x10^{-01}$	[6]
BBS4	212745_s_at	Bardet-Biedl syndrome 4	100	-1.19	$1.27x10^{-01}$	[6]
BBS5	230697_at	Bardet-Biedl syndrome 5	94	-1.36	$6.36x10^{-03}$	[7]
BBS7	219688_at	Bardet-Biedl syndrome 7	88	-1.03	$8.92x10^{-01}$	[8]
BBS7	235007_at	Bardet-Biedl syndrome 7	100	-1.15	$2.80x10^{-01}$	[8]
CDKN3	1555758_a_at	cyclin-dependent kinase inhibitor 3	6	1.14	$7.78x10^{-01}$	[9]
CDKN3	209714_s_at	cyclin-dependent kinase inhibitor 3	12	1.21	$4.86x10^{-01}$	[9]
CEP164	1558953_s_at	centrosomal protein 164kDa	100	-1.05	$7.48x10^{-01}$	[10]
CEP164	204250_s_at	centrosomal protein 164kDa	69	-1.14	$5.42x10^{-01}$	[10]
CEP164	204251_s_at	centrosomal protein 164kDa	100	1.00	$9.87x10^{-01}$	[10]
CETN2	209194_at	centrin, EF-hand protein, 2	100	-1.17	$8.74x10^{-02}$	[11]
CRB3	232609_at	crumbs homolog 3 (Drosophila)	75	-1.02	$9.29x10^{-01}$	[12]
CROCC	206274_s_at	ciliary rootlet coiled-coil, rootletin	100	-1.17	$4.86x10^{-01}$	[12]
CROCC	216419_at	ciliary rootlet coiled-coil, rootletin	12	-1.31	$3.65x10^{-01}$	[12]
DNAHx10	1562462_at	dynein, axonemal, heavy chain x10	92	-1.75	$2.40x10^{-03}$	[13]
DNAHx10	1565339_at	dynein, axonemal, heavy chain x10	65	-1.94	$3.24x10^{-03}$	[13]
DNAHx10	229738_at	dynein, axonemal, heavy chain x10	100	-1.26	$5.76x10^{-02}$	[13]
DNAH11	1553159_at	dynein, axonemal, heavy chain 11	98	-1.57	$9.03x10^{-04}$	[14]
DNAH11	1560416_at	dynein, axonemal, heavy chain 11	100	-1.04	$8.35x10^{-01}$	[14]

Supplemental Table II: Cilia-related Gene Expression in Airway Epithelium [1] (cont., page 2)

Gene symbol	Probe set ID	Gene title	P call (%)	Fold-change[2]	p value[3]	Reference
DNAH11	237804_at	Axonemal dynein heavy chain (DNAH11), partial	43	-1.00	$9.98x10^{-01}$	[14]
DNAH3	1560803_at	dynein, axonemal, heavy chain 3	96	-1.08	$6.34x10^{-01}$	[15]
DNAH3	1563290_at	dynein, axonemal, heavy chain 3	29	-1.43	$7.47x10^{-02}$	[15]
DNAH3	215266_at	dynein, axonemal, heavy chain 3	100	-1.36	$5.24x10^{-02}$	[15]
DNAH3	216415_at	dynein, axonemal, heavy chain 3 (DNAH3), mRNA	71	-1.00	$9.86x10^{-01}$	[15]
DNAH3	220725__at	dynein, axonemal, heavy chain 3 (DNAH3), mRNA	100	-1.06	$7.19x10^{-01}$	[15]
DNAH5	232381_s_at	dynein, axonemal, heavy chain 5	100	-1.68	$9.60x10^{-05}$	[16]
DNAH5	243938__at	dynein, axonemal, heavy chain 5	100	-1.83	$9.60x10^{-05}$	[16]
DNAH7	214222_at	dynein, axonemal, heavy chain 7	100	-1.31	$4.79x10^{-03}$	[17]
DNAH7	234476_at	dynein, axonemal, heavy chain 7	100	-1.23	$8.85x10^{-02}$	[17]
DNAH7	234727_at	dynein, axonemal, heavy chain 7	29	1.37	$2.x10x10^{-01}$	[17]
DNAH9	207959_s_at	dynein, axonemal, heavy chain 9	100	-1.19	$1.67x10^{-01}$	[18]
DNAH9	2x10345_s_at	dynein, axonemal, heavy chain 9	100	-1.56	$1.40x10^{-02}$	[18]
DNAH9	240857_at	dynein, axonemal, heavy chain 9	100	-1.39	$3.24x10^{-03}$	[18]
DNAI1	220125_at	dynein, axonemal, intermediate chain 1	100	-1.06	$7.48x10^{-01}$	[19]
DNAI1	233195_at	dynein, axonemal, intermediate chain 1	59	1.09	$4.88x10^{-01}$	[19]
DNAI2	220636_at	dynein, axonemal, intermediate chain 2	100	1.07	$5.42x10^{-01}$	[20]
DNAI2	221668_s_at	dynein, axonemal, intermediate chain 2	100	-1.07	$7.40x10^{-01}$	[20]
DNAL4	204008_at	dynein, axonemal, light chain 4	100	-1.06	$6.95x10^{-01}$	[21]
DNALI1	205186_at	dynein, axonemal, light intermediate chain 1	100	-1.46	$3.12x10^{-02}$	[22]
DNALI1	227081_at	dynein, axonemal, light intermediate chain 1	100	-1.21	$5.58x10^{-02}$	[22]
DYNC1H1	1556831_at	KIAA0325 protein	29	1.11	$7.65x10^{-01}$	[23]
DYNC1H1	211928_at	dynein, cytoplasmic 1, heavy chain 1	100	-1.05	$7.48x10^{-01}$	[23]
DYNC1H1	229042_s_at	dynein, cytoplasmic 1, heavy chain 1	2	1.47	$3.69x10^{-01}$	[23]
DYNC1H1	229115_at	dynein, cytoplasmic 1, heavy chain 1	20	-1.03	$9.20x10^{-01}$	[23]
DYNC1H1	236183_at	dynein, cytoplasmic 1, heavy chain 1	0	1.16	$7.90x10^{-01}$	[23]
DYNC1H1	24x1084__at	dynein, cytoplasmic 1, heavy chain 1	0	-1.09	$8.85x10^{-01}$	[23]
DYNC1H1	244768_at	dynein, cytoplasmic 1, heavy chain 1	0	-1.05	$9.20x10^{-01}$	[23]
DYNC1I2	211684_s_at	dynein, cytoplasmic 1, intermediate chain 2	100	-1.08	$4.51x10^{-01}$	[24]
DYNC2H1	1561939_at	dynein, cytoplasmic 2, heavy chain 1	100	-1.90	$9.03x10^{-04}$	[25]
DYNC2H1	1565149_at	dynein, cytoplasmic 2, heavy chain 1	100	1.05	$7.89x10^{-01}$	[25]
DYNC2H1	219469_at	dynein, cytoplasmic 2, heavy chain 1	100	-1.26	$6.63x10^{-02}$	[25]

Supplemental Table II: Cilia-related Gene Expression in Airway Epithelium [1] (cont., page 3)

Gene symbol	Probe set ID	Gene title	P call (%)	Fold-change[2]	p value[3]	Reference
DYNC2LI1	1554324_s_at	dynein, cytoplasmic 2, light intermediate chain 1	82	-1.03	$8.92x10^{-01}$	[26]
DYNC2LI1	203762_s_at	dynein, cytoplasmic 2, light intermediate chain 1	100	-1.04	$7.97x10^{-01}$	[26]
DYNC2LI1	203763_at	dynein, cytoplasmic 2, light intermediate chain 1	100	-1.14	$2.85x10^{-01}$	[26]
DYNC2LI1	241757__at	dynein, cytoplasmic 2, light intermediate chain 1	100	-1.01	$9.58x10^{-01}$	[26]
DYNLT1	201999_s_at	dynein, light chain, Tcte-type 1	100	-1.08	$3.42x10^{-01}$	[27]
EFHC1	219833_s_at	EF-hand domain (C-terminal) containing 1	100	-1.18	$5.87x10^{-02}$	[28]
EFHC1	225656_at	EF-hand domain (C-terminal) containing 1	100	-1.05	$7.19x10^{-01}$	[28]
EFHC1	23x1026_at	EF-hand domain (C-terminal) containing 1	100	-1.24	$3.12x10^{-02}$	[28]
EML1	204796_at	echinoderm microtubule associated protein like 1	100	-1.41	$2.48x10^{-02}$	[29]
EML1	204797_s_at	echinoderm microtubule associated protein like 1	100	-1.36	$3.24x10^{-03}$	[29]
EZR	208621_s_at	ezrin	100	-1.67	$5.64x10^{-03}$	[30]
EZR	208622_s_at	ezrin	100	-1.41	$1.02x10^{-02}$	[30]
EZR	208623_s_at	ezrin	100	-1.15	$3.88x10^{-02}$	[30]
EZR	217230_at	ezrin	49	1.09	$8.52x10^{-01}$	[30]
EZR	217234_s_at	ezrin	100	-1.69	$4.47x10^{-03}$	[30]
F2RL2	206795_at	coagulation factor II (thrombin) receptor-like 2	0	1.24	$6.79x10^{-01}$	[31]
F2RL2	230147_at	coagulation factor II (thrombin) receptor-like 2	6	2.60	$1.38x10^{-02}$	[31]
FOXJ1	205906_at	forkhead box J1	100	-1.38	$1.53x10^{-01}$	[30]
GAS8	204921_at	growth arrest-specific 8	100	1.01	$9.33x10^{-01}$	[32]
GSK3B	209945_s_at	glycogen synthase kinase 3 beta	100	-1.13	$2.78x10^{-01}$	[33]
IFT122	1563794_s_at	intraflagellar transport 122 homolog (Chlamydomonas)	51	-1.01	$9.75x10^{-01}$	[34]
IFT122	216678_at	intraflagellar transport 122 homolog (Chlamydomonas)	73	-1.06	$7.89x10^{-01}$	[34]
IFT122	220744_s_at	intraflagellar transport 122 homolog (Chlamydomonas)	100	-1.02	$8.77x10^{-01}$	[34]
IFT140	204792_s_at	intraflagellar transport 140 homolog (Chlamydomonas)	100	1.06	$7.89x10^{-01}$	[35]
IFT140	232844_at	KIAA0590 protein	43	1.03	$8.52x10^{-01}$	[35]
IFT140	244585_at	intraflagellar transport 140 homolog (Chlamydomonas)	0	1.42	$3.65x10^{-01}$	[35]
IFT172	226324_s_at	intraflagellar transport 172 homolog (Chlamydomonas)	100	-1.24	$1.50x10^{-02}$	[36]
IFT20	2x10312_s_at	intraflagellar transport 20 homolog (Chlamydomonas)	100	-1.22	$4.75x10^{-02}$	[37]
IFT52	218709_s_at	intraflagellar transport 52 homolog (Chlamydomonas)	100	-1.17	$6.24x10^{-02}$	[38]
IFT52	233532__at	intraflagellar transport 52 homolog (Chlamydomonas)	92	-1.19	$1.82x10^{-01}$	[38]
IFT57	218x100_s_at	intraflagellar transport 57 homolog (Chlamydomonas)	100	-1.17	$1.41x10^{-01}$	[37]
IFT57	222519_s_at	intraflagellar transport 57 homolog (Chlamydomonas)	100	-1.46	$3.12x10^{-02}$	[37]

Supplemental Table II: Cilia-related Gene Expression in Airway Epithelium [1] (cont., page 4)

Gene symbol	Probe set ID	Gene title	P call (%)	Fold-change[2]	p value[3]	Reference
IFT57	222520_s_at	intraflagellar transport 57 homolog (Chlamydomonas)	100	-1.46	$2.40x10^{-03}$	[37]
IFT80	1558956_s_at	intraflagellar transport 80 homolog (Chlamydomonas)	100	-1.03	$8.92x10^{-01}$	[39]
IFT80	1564231_at	intraflagellar transport 80 homolog (Chlamydomonas)	96	1.37	$5.94x10^{-03}$	[39]
IFT80	226098_at	intraflagellar transport 80 homolog (Chlamydomonas)	100	-1.08	$5.42x10^{-01}$	[39]
IFT80	240251_at	CDNA FLJ52072 complete cds, highly similar to Intraflagellar transport 80 homolog	22	1.67	$9.61x10^{-02}$	[39]
IFT88	204703_at	intraflagellar transport 88 homolog (Chlamydomonas)	100	-1.18	$5.82x10^{-02}$	[13]
KIF17	222144_at	kinesin family member 17	18	-1.07	$8.77x10^{-01}$	[40]
KIF3A	213623_at	kinesin family member 3A	100	-1.28	$5.55x10^{-02}$	[23]
KIF3A	228680_at	kinesin family member 3A	100	-1.26	$8.52x10^{-03}$	[23]
KIF3B	203943_at	kinesin family member 3B	100	-1.01	$9.29x10^{-01}$	[23]
KIF3B	225205_at	kinesin family member 3B	100	-1.05	$6.25x10^{-01}$	[23]
KIFAP3	203333_at	kinesin-associated protein 3	100	-1.24	$5.24x10^{-02}$	[41]
KLC1	212877_at	kinesin light chain 1	98	1.06	$6.34x10^{-01}$	[42]
KLC1	212878_s_at	kinesin light chain 1	100	-1.23	$6.29x10^{-02}$	[42]
KLC1	213656_s_at	kinesin light chain 1	100	-1.03	$7.89x10^{-01}$	[42]
KLC1	232036_at	KLC1 mRNA for kinesin light chain 1, complete cds, clone: FLJ08153AAAN	2	1.14	$8.09x10^{-01}$	[42]
KLC2	218906__at	kinesin light chain 2	65	-1.04	$8.52x10^{-01}$	[42]
LBR	201795_at	lamin B receptor	100	-1.x10	$5.57x10^{-01}$	[43]
MKS1	1555820_a_at	Meckel syndrome, type 1	100	-1.20	$1.41x10^{-01}$	[44]
MKS1	218630_at	Meckel syndrome, type 1	100	-1.08	$5.42x10^{-01}$	[44]
MLF1	204783_at	myeloid leukemia factor 1	100	-1.38	$4.05x10^{-03}$	[32]
MLF1	204784_s_at	myeloid leukemia factor 1	100	-1.41	$3.24x10^{-03}$	[32]
MYCBP	203359_s_at	c-myc binding protein	100	1.03	$8.52x10^{-01}$	[32]
MYCBP	203360_s_at	c-myc binding protein	100	-1.14	$2.60x10^{-01}$	[32]
MYCBP	203361_s_at	c-myc binding protein	100	-1.01	$9.54x10^{-01}$	[32]
NASP	201969_at	nuclear autoantigenic sperm protein (histone-binding)	94	-1.09	$4.65x10^{-01}$	[45]
NASP	201970_s_at	nuclear autoantigenic sperm protein (histone-binding)	100	-1.29	$4.75x10^{-02}$	[45]
NASP	237276_at	Nuclear autoantigenic sperm protein (histone-binding) (NASP), transcript variant 1, mRNA	0	1.03	$9.38x10^{-01}$	[45]
NASP	242918_at	Nuclear autoantigenic sperm protein (histone-binding)	65	-1.23	$4.29x10^{-01}$	[45]

Supplemental Table II: Cilia-related Gene Expression in Airway Epithelium [1] (cont., page 5)

Gene symbol	Probe set ID	Gene title	P call (%)	Fold-change[2]	p value[3]	Reference
		(NASP), transcript variant 1, mRNA				
NME5	206197_at	non-metastatic cells 5, protein epressed in (nucleoside-diphosphate kinase)	100	-1.14	$2.x10x10^{-01}$	[32]
NME7	219553_at	non-metastatic cells 7, protein epressed in (nucleoside-diphosphate kinase)	100	-1.x10	$4.77x10^{-01}$	[32]
NME7	227556_at	non-metastatic cells 7, protein epressed in (nucleoside-diphosphate kinase)	100	1.x10	$7.04x10^{-01}$	[32]
NPHP1	206285_at	nephronophthisis 1 (juvenile)	78	1.11	$5.42x10^{-01}$	[46]
NPHP1	238843_at	nephronophthisis 1 (juvenile)	100	1.02	$9.20x10^{-01}$	[46]
NPHP1	238844_s_at	nephronophthisis 1 (juvenile)	100	-1.41	$4.79x10^{-03}$	[46]
NPHP3	1553389_at	nephronophthisis 3 (adolescent)	16	1.11	$7.97x10^{-01}$	[46]
NPHP4	213471_at	nephronophthisis 4	98	1.09	$5.57x10^{-01}$	[46]
NPHP4	216344_at	nephronophthisis 4	6	1.05	$9.20x10^{-01}$	[46]
ODF2	2x10415_s_at	outer dense fiber of sperm tails 2	82	-1.63	$4.05x10^{-03}$	[47]
ODF2	225617_at	outer dense fiber of sperm tails 2	94	-1.17	$5.37x10^{-01}$	[47]
OFD1	203569_s_at	oral-facial-digital syndrome 1	100	-1.18	$3.57x10^{-02}$	[48]
OFD1	241751_at	oral-facial-digital syndrome 1	100	1.x10	$7.18x10^{-01}$	[48]
PACRG	214204_at	PARK2 co-regulated	100	-1.17	$2.31x10^{-01}$	[49]
PACRG	215472_at	PARK2 co-regulated	100	-1.25	$7.47x10^{-02}$	[49]
PCM1	202174_s_at	pericentriolar material 1	100	-1.28	$4.79x10^{-03}$	[50]
PCM1	209996__at	pericentriolar material 1	14	-1.53	$5.76x10^{-02}$	[50]
PCM1	209997__at	pericentriolar material 1	100	-1.20	$1.24x10^{-01}$	[50]
PCM1	214118__at	pericentriolar material 1	100	-1.08	$6.14x10^{-01}$	[50]
PCM1	214937__at	pericentriolar material 1	100	-1.09	$2.21x10^{-01}$	[50]
PCM1	228905_at	pericentriolar material 1	100	-1.37	$9.44x10^{-03}$	[50]
PCNT	203660_s_at	pericentrin	98	-1.05	$7.09x10^{-01}$	[51]
PCNT	233387_s_at	pericentrin	14	-1.71	$5.34x10^{-02}$	[51]
PKD1	202327_s_at	polycystic kidney disease 1 (autosomal dominant)	25	-1.17	$5.68x10^{-01}$	[52]
PKD1	202328_s_at	polycystic kidney disease 1 (autosomal dominant)	98	-1.01	$9.58x10^{-01}$	[52]
PKD1	216949_s_at	polycystic kidney disease 1 (autosomal dominant)	25	-1.47	$3.71x10^{-01}$	[52]
PKD2	203688_at	polycystic kidney disease 2 (autosomal dominant)	100	-1.31	$3.71x10^{-02}$	[52]
PLEKHA8	1560556_a_at	Homo sapiens, clone IMAGE:4617677, mRNA	20	1.22	$7.19x10^{-01}$	[53]

Supplemental Table II: Cilia-related Gene Expression in Airway Epithelium [1] (cont., page 6)

Gene symbol	Probe set ID	Gene title	P call (%)	Fold-change[2]	p value[3]	Reference
PLEKHA8	224208_at	pleckstrin homology domain containing, family A (phosphoinositide binding specific) member 8	2	1.27	6.85×10^{-01}	[53]
PLEKHA8	231543_at	pleckstrin homology domain containing, family A (phosphoinositide binding specific) member 8	0	-1.x10	8.52×10^{-01}	[53]
PLEKHA8	232212_at	pleckstrin homology domain containing, family A (phosphoinositide binding specific) member 8	2	-1.08	8.62×10^{-01}	[53]
PLEKHA8	233943__at	pleckstrin homology domain containing, family A (phosphoinositide binding specific) member 8	47	1.34	2.86×10^{-01}	[53]
PLEKHA8 /// PLEKHA9	220157__at	pleckstrin homology domain containing, family A (phosphoinositide binding specific) member 8 /// pleckstrin homology domain containing, family A (phosphoinositide binding specific) member 9	61	1.08	6.64×10^{-01}	[53]
PPP2R1A	200695_at	protein phosphatase 2 (formerly 2A), regulatory subunit A, alpha isoform	96	-1.20	2.78×10^{-01}	[32]
RABL4	205037_at	RAB, member of RAS oncogene family-like 4	100	-1.21	1.27×10^{-01}	[32]
RABL4	213784_at	RAB, member of RAS oncogene family-like 4	100	-1.04	8.32×10^{-01}	[32]
RABL4	243812_at	RAB, member of RAS oncogene family-like 4	0	-1.03	9.45×10^{-01}	[32]
RPGRIP1L	213959_s_at	RPGRIP1-like	100	-1.02	9.20×10^{-01}	[54]
RPGRIP1L	24x1028_at	RPGRIP1-like, mRNA (cDNA clone IMAGE:4697938)	6	-1.04	9.29×10^{-01}	[54]
RSPH3	1568613_at	radial spoke 3 homolog (Chlamydomonas)	0	-1.03	8.86×10^{-01}	[55]
RSPH3	223713_at	radial spoke 3 homolog (Chlamydomonas)	98	-1.16	3.90×10^{-01}	[55]
SLC9A3R1	201349_at	solute carrier family 9 (sodium/hydrogen echanger), member 3 regulator 1	100	-1.08	5.18×10^{-01}	[56]
SPAG6	2x10032_s_at	sperm associated antigen 6	100	-1.45	2.48×10^{-03}	[57]
SPAG6	2x10033_s_at	sperm associated antigen 6	100	-1.34	3.31×10^{-03}	[57]
TCTE3	1554400_at	t-comple-associated-testis-epressed 3	8	1.63	$2.x10 \times 10^{-01}$	[58]
TCTE3	1554401_a_at	t-comple-associated-testis-epressed 3	2	-1.40	4.74×10^{-01}	[58]
TCTE3	1557945_at	dynein light chain 2 TCTE2	98	1.05	7.48×10^{-01}	[58]
TCTE3	232258_at	dynein light chain 2 TCTE2	4	-1.01	9.77×10^{-01}	[58]
TEKT1	239216_at	tektin 1	100	-1.45	2.40×10^{-03}	[59]
TEKT2	2x10323_at	tektin 2 (testicular)	100	-1.37	3.77×10^{-02}	[60]
TEKT3	223867_at	tektin 3	100	-1.27	5.87×10^{-02}	[61]

Supplemental Table II: Cilia-related Gene Expression in Airway Epithelium [1] (cont., page 7)

Gene symbol	Probe set ID	Gene title	P call (%)	Fold-change[2]	p value[3]	Reference
TMEM67	1552763_at	transmembrane protein 67	14	-1.58	$3.42x10^{-01}$	[44]
TMEM67	1552765__at	transmembrane protein 67	37	-1.05	$9.29x10^{-01}$	[44]
TMEM67	1563646_a_at	transmembrane protein 67	100	-1.44	$3.12x10^{-02}$	[44]
TMEM67	1569377_at	transmembrane protein 67	100	-1.02	$9.29x10^{-01}$	[44]
TMEM67	1569411_at	transmembrane protein 67	22	-1.57	$2.28x10^{-01}$	[44]
TMEM67	232023_at	transmembrane protein 67	100	-1.38	$4.75x10^{-02}$	[44]
TMEM67	238229_at	transmembrane protein 67	100	-1.17	$2.31x10^{-01}$	[44]
TUBA1C	209251__at	tubulin, alpha 1c	100	-1.01	$9.33x10^{-01}$	[62]
TUBA1C	211750__at	tubulin, alpha 1c	100	-1.03	$7.90x10^{-01}$	[62]
TUBB2A	204141_at	tubulin, beta 2A	100	-1.08	$6.44x10^{-01}$	[62]
TUBB2A /// TUBB2B	209372__at	tubulin, beta 2A /// tubulin, beta 2B	90	-1.64	$4.75x10^{-02}$	[62]
VCP	208648_at	valosin-containing protein	100	-1.01	$9.29x10^{-01}$	[32,63]
VCP	208649_s_at	valosin-containing protein	100	-1.04	$7.60x10^{-01}$	[32,63]
VHL	1559227_s_at	von Hippel-Lindau tumor suppressor	59	1.05	$7.90x10^{-01}$	[64]
VHL	203844_at	von Hippel-Lindau tumor suppressor	29	1.53	$4.19x10^{-01}$	[64]

[1] The HG-U133 Plus 2.0 microarray platform was used to detect differences in the expression of these genes in healthy smokers compared to healthy nonsmokers.

[2] Magnitude of fold change in average expression values for healthy smokers *vs* healthy nonsmokers.

[3] A Benjamini-Hochberg correction was used to limit the false positive rate.

References

1. Kultgen PL, Byrd SK, Ostrowski LE, Milgram SL (2002) Characterization of an A-kinase anchoring protein in human ciliary axonemes. Mol Biol Cell 13: 4156-4166.
2. Hearn T, Spalluto C, Phillips VJ, Renforth GL, Copin N, Hanley NA, Wilson DI (2005) Subcellular localization of ALMS1 supports involvement of centrosome and basal body dysfunction in the pathogenesis of obesity, insulin resistance, and type 2 diabetes. Diabetes 54: 1581-1587.
3. Kulaga HM, Leitch CC, Eichers ER, Badano JL, Lesemann A, Hoskins BE, Lupski JR, Beales PL, Reed RR, Katsanis N (2004) Loss of BBS proteins causes anosmia in humans and defects in olfactory cilia structure and function in the mouse. Nat Genet 36: 994-998.
4. Nachury MV, Loktev AV, Zhang Q, Westlake CJ, Peranen J, Merdes A, Slusarski DC, Scheller RH, Bazan JF, Sheffield VC, Jackson PK (2007) A core complex of BBS proteins cooperates with the GTPase Rab8 to promote ciliary membrane biogenesis. Cell 129: 1201-1213.
5. Nishimura DY, Fath M, Mullins RF, Searby C, Andrews M, Davis R, Andorf JL, Mykytyn K, Swiderski RE, Yang B, Carmi R, Stone EM, Sheffield VC (2004) Bbs2-null mice have neurosensory deficits, a defect in social dominance, and retinopathy associated with mislocalization of rhodopsin. Proc Natl Acad Sci U S A 101: 16588-16593.
6. Kim JC, Badano JL, Sibold S, Esmail MA, Hill J, Hoskins BE, Leitch CC, Venner K, Ansley SJ, Ross AJ, Leroux MR, Katsanis N, Beales PL (2004) The Bardet-Biedl protein BBS4 targets cargo to the pericentriolar region and is required for microtubule anchoring and cell cycle progression. Nat Genet 36: 462-470.

7. Li JB, Gerdes JM, Haycraft CJ, Fan Y, Teslovich TM, May-Simera H, Li H, Blacque OE, Li L, Leitch CC, Lewis RA, Green JS, Parfrey PS, Leroux MR, Davidson WS, Beales PL, Guay-Woodford LM, Yoder BK, Stormo GD, Katsanis N, Dutcher SK (2004) Comparative genomics identifies a flagellar and basal body proteome that includes the BBS5 human disease gene. Cell 117: 541-552.

8. Blacque OE, Reardon MJ, Li C, McCarthy J, Mahjoub MR, Ansley SJ, Badano JL, Mah AK, Beales PL, Davidson WS, Johnsen RC, Audeh M, Plasterk RH, Baillie DL, Katsanis N, Quarmby LM, Wicks SR, Leroux MR (2004) Loss of C. elegans BBS-7 and BBS-8 protein function results in cilia defects and compromised intraflagellar transport. Genes Dev 18: 1630-1642.

9. Scholey JM (2008) Intraflagellar transport motors in cilia: moving along the cell's antenna. J Cell Biol 180: 23-29.

10. Graser S, Stierhof YD, Lavoie SB, Gassner OS, Lamla S, Le CM, Nigg EA (2007) Cep164, a novel centriole appendage protein required for primary cilium formation. J Cell Biol 179: 321-330.

11. Laoukili J, Perret E, Middendorp S, Houcine O, Guennou C, Marano F, Bornens M, Tournier F (2000) Differential expression and cellular distribution of centrin isoforms during human ciliated cell differentiation in vitro. J Cell Sci 113 (Pt 8): 1355-1364.

12. Yang J, Liu X, Yue G, Adamian M, Bulgakov O, Li T (2002) Rootletin, a novel coiled-coil protein, is a structural component of the ciliary rootlet. J Cell Biol 159: 431-440.

13. Pazour GJ, Agrin N, Walker BL, Witman GB (2006) Identification of predicted human outer dynein arm genes: candidates for primary ciliary dyskinesia genes. J Med Genet 43: 62-73.

14. Schwabe GC, Hoffmann K, Loges NT, Birker D, Rossier C, de Santi MM, Olbrich H, Fliegauf M, Failly M, Liebers U, Collura M, Gaedicke G, Mundlos S, Wahn U, Blouin JL, Niggemann B, Omran H, Antonarakis SE, Bartoloni L (2008) Primary ciliary dyskinesia associated with normal axoneme ultrastructure is caused by DNAH11 mutations. Hum Mutat 29: 289-298.

15. Chapelin C, Duriez B, Magnino F, Goossens M, Escudier E, Amselem S (1997) Isolation of several human axonemal dynein heavy chain genes: genomic structure of the catalytic site, phylogenetic analysis and chromosomal assignment. FEBS Lett 412: 325-330.

16. Hornef N, Olbrich H, Horvath J, Zariwala MA, Fliegauf M, Loges NT, Wildhaber J, Noone PG, Kennedy M, Antonarakis SE, Blouin JL, Bartoloni L, Nusslein T, Ahrens P, Griese M, Kuhl H, Sudbrak R, Knowles MR, Reinhardt R, Omran H (2006) DNAH5 mutations are a common cause of primary ciliary dyskinesia with outer dynein arm defects. Am J Respir Crit Care Med 174: 120-126.

17. Zhang YJ, O'Neal WK, Randell SH, Blackburn K, Moyer MB, Boucher RC, Ostrowski LE (2002) Identification of dynein heavy chain 7 as an inner arm component of human cilia that is synthesized but not assembled in a case of primary ciliary dyskinesia. J Biol Chem 277: 17906-17915.

18. Fliegauf M, Olbrich H, Horvath J, Wildhaber JH, Zariwala MA, Kennedy M, Knowles MR, Omran H (2005) Mislocalization of DNAH5 and DNAH9 in respiratory cells from patients with primary ciliary dyskinesia. Am J Respir Crit Care Med 171: 1343-1349.

19. Zariwala MA, Leigh MW, Ceppa F, Kennedy MP, Noone PG, Carson JL, Hazucha MJ, Lori A, Horvath J, Olbrich H, Loges NT, Bridoux AM, Pennarun G, Duriez B, Escudier E, Mitchison HM, Chodhari R, Chung EM, Morgan LC, de Iongh RU, Rutland J, Pradal U,

Omran H, Amselem S, Knowles MR (2006) Mutations of DNAI1 in primary ciliary dyskinesia: evidence of founder effect in a common mutation. Am J Respir Crit Care Med 174: 858-866.

20. Loges NT, Olbrich H, Fenske L, Mussaffi H, Horvath J, Fliegauf M, Kuhl H, Baktai G, Peterffy E, Chodhari R, Chung EM, Rutman A, O'Callaghan C, Blau H, Tiszlavicz L, Voelkel K, Witt M, Zietkiewicz E, Neesen J, Reinhardt R, Mitchison HM, Omran H (2008) DNAI2 mutations cause primary ciliary dyskinesia with defects in the outer dynein arm. Am J Hum Genet 83: 547-558.

21. Iwasaki M, Kuwata T, Yamazaki Y, Jenkins NA, Copeland NG, Osato M, Ito Y, Kroon E, Sauvageau G, Nakamura T (2005) Identification of cooperative genes for NUP98-HOXA9 in myeloid leukemogenesis using a mouse model. Blood 105: 784-793.

22. Rashid S, Breckle R, Hupe M, Geisler S, Doerwald N, Neesen J (2006) The murine Dnali1 gene encodes a flagellar protein that interacts with the cytoplasmic dynein heavy chain 1. Mol Reprod Dev 73: 784-794.

23. Rosenbaum JL, Witman GB (2002) Intraflagellar transport. Nat Rev Mol Cell Biol 3: 813-825.

24. Vaughan KT, Vallee RB (1995) Cytoplasmic dynein binds dynactin through a direct interaction between the intermediate chains and p150Glued. J Cell Biol 131: 1507-1516.

25. Perrone CA, Tritschler D, Taulman P, Bower R, Yoder BK, Porter ME (2003) A novel dynein light intermediate chain colocalizes with the retrograde motor for intraflagellar transport at sites of axoneme assembly in chlamydomonas and Mammalian cells. Mol Biol Cell 14: 2041-2056.

26. Grissom PM, Vaisberg EA, McIntosh JR (2002) Identification of a novel light intermediate chain (D2LIC) for mammalian cytoplasmic dynein 2. Mol Biol Cell 13: 817-829.

27. Pfister KK, Shah PR, Hummerich H, Russ A, Cotton J, Annuar AA, King SM, Fisher EM (2006) Genetic analysis of the cytoplasmic dynein subunit families. PLoS Genet 2: e1.

28. Ikeda T, Ikeda K, Enomoto M, Park MK, Hirono M, Kamiya R (2005) The mouse ortholog of EFHC1 implicated in juvenile myoclonic epilepsy is an axonemal protein widely conserved among organisms with motile cilia and flagella. FEBS Lett 579: 819-822.

29. Hueston JL, Herren GP, Cueva JG, Buechner M, Lundquist EA, Goodman MB, Suprenant KA (2008) The C. elegans EMAP-like protein, ELP-1 is required for touch sensation and associates with microtubules and adhesion complexes. BMC Dev Biol 8: 110.

30. Gomperts BN, Belperio JA, Fishbein MC, Keane MP, Burdick MD, Strieter RM (2007) Keratinocyte growth factor improves repair in the injured tracheal epithelium. Am J Respir Cell Mol Biol 37: 48-56.

31. Sfakianos J, Togawa A, Maday S, Hull M, Pypaert M, Cantley L, Toomre D, Mellman I (2007) Par3 functions in the biogenesis of the primary cilium in polarized epithelial cells. J Cell Biol 179: 1133-1140.

32. Ostrowski LE, Blackburn K, Radde KM, Moyer MB, Schlatzer DM, Moseley A, Boucher RC (2002) A proteomic analysis of human cilia: identification of novel components. Mol Cell Proteomics 1: 451-465.

33. Wilson NF, Lefebvre PA (2004) Regulation of flagellar assembly by glycogen synthase kinase 3 in Chlamydomonas reinhardtii. Eukaryot Cell 3: 1307-1319.

34. Eggenschwiler JT, Anderson KV (2007) Cilia and developmental signaling. Annu Rev Cell Dev Biol 23: 345-373.

35. Tsujikawa M, Malicki J (2004) Intraflagellar transport genes are essential for differentiation and survival of vertebrate sensory neurons. Neuron 42: 703-716.

36. Huangfu D, Liu A, Rakeman AS, Murcia NS, Niswander L, Anderson KV (2003) Hedgehog signalling in the mouse requires intraflagellar transport proteins. Nature 426: 83-87.

37. Follit JA, Tuft RA, Fogarty KE, Pazour GJ (2006) The intraflagellar transport protein IFT20 is associated with the Golgi complex and is required for cilia assembly. Mol Biol Cell 17: 3781-3792.

38. Brazelton WJ, Amundsen CD, Silflow CD, Lefebvre PA (2001) The bld1 mutation identifies the Chlamydomonas osm-6 homolog as a gene required for flagellar assembly. Curr Biol 11: 1591-1594.

39. Beales PL, Bland E, Tobin JL, Bacchelli C, Tuysuz B, Hill J, Rix S, Pearson CG, Kai M, Hartley J, Johnson C, Irving M, Elcioglu N, Winey M, Tada M, Scambler PJ (2007) IFT80, which encodes a conserved intraflagellar transport protein, is mutated in Jeune asphyxiating thoracic dystrophy. Nat Genet 39: 727-729.

40. Jenkins PM, Hurd TW, Zhang L, McEwen DP, Brown RL, Margolis B, Verhey KJ, Martens JR (2006) Ciliary targeting of olfactory CNG channels requires the CNGB1b subunit and the kinesin-2 motor protein, KIF17. Curr Biol 16: 1211-1216.

41. Liu Q, Tan G, Levenkova N, Li T, Pugh EN, Jr., Rux JJ, Speicher DW, Pierce EA (2007) The proteome of the mouse photoreceptor sensory cilium complex. Mol Cell Proteomics 6: 1299-1317.

42. Yang J, Li T (2005) The ciliary rootlet interacts with kinesin light chains and may provide a scaffold for kinesin-1 vesicular cargos. Exp Cell Res 309: 379-389.

43. Mylonis I, Drosou V, Brancorsini S, Nikolakaki E, Sassone-Corsi P, Giannakouros T (2004) Temporal association of protamine 1 with the inner nuclear membrane protein lamin B receptor during spermiogenesis. J Biol Chem 279: 11626-11631.

44. Dawe HR, Smith UM, Cullinane AR, Gerrelli D, Cox P, Badano JL, Blair-Reid S, Sriram N, Katsanis N, ttie-Bitach T, Afford SC, Copp AJ, Kelly DA, Gull K, Johnson CA (2007) The Meckel-Gruber Syndrome proteins MKS1 and meckelin interact and are required for primary cilium formation. Hum Mol Genet 16: 173-186.

45. Welch JE, O'Rand MG (1990) Characterization of a sperm-specific nuclear autoantigenic protein. II. Expression and localization in the testis. Biol Reprod 43: 569-578.

46. Otto EA, Schermer B, Obara T, O'Toole JF, Hiller KS, Mueller AM, Ruf RG, Hoefele J, Beekmann F, Landau D, Foreman JW, Goodship JA, Strachan T, Kispert A, Wolf MT, Gagnadoux MF, Nivet H, Antignac C, Walz G, Drummond IA, Benzing T, Hildebrandt F (2003) Mutations in INVS encoding inversin cause nephronophthisis type 2, linking renal cystic disease to the function of primary cilia and left-right axis determination. Nat Genet 34: 413-420.

47. Ishikawa H, Kubo A, Tsukita S, Tsukita S (2005) Odf2-deficient mother centrioles lack distal/subdistal appendages and the ability to generate primary cilia. Nat Cell Biol 7: 517-524.

48. Ferrante MI, Zullo A, Barra A, Bimonte S, Messaddeq N, Studer M, Dolle P, Franco B (2006) Oral-facial-digital type I protein is required for primary cilia formation and left-right axis specification. Nat Genet 38: 112-117.

49. Dawe HR, Farr H, Portman N, Shaw MK, Gull K (2005) The Parkin co-regulated gene product, PACRG, is an evolutionarily conserved axonemal protein that functions in outer-doublet microtubule morphogenesis. J Cell Sci 118: 5421-5430.

50. Vladar EK, Stearns T (2007) Molecular characterization of centriole assembly in ciliated epithelial cells. J Cell Biol 178: 31-42.

51. Jurczyk A, Gromley A, Redick S, San AJ, Witman G, Pazour GJ, Peters DJ, Doxsey S (2004) Pericentrin forms a complex with intraflagellar transport proteins and polycystin-2 and is required for primary cilia assembly. J Cell Biol 166: 637-643.

52. Ong AC, Wheatley DN (2003) Polycystic kidney disease--the ciliary connection. Lancet 361: 774-776.

53. De Matteis MA, Di CA, D'Angelo G (2007) Lipid-transfer proteins in membrane trafficking at the Golgi complex. Biochim Biophys Acta 1771: 761-768.

54. Arts HH, Doherty D, van Beersum SE, Parisi MA, Letteboer SJ, Gorden NT, Peters TA, Marker T, Voesenek K, Kartono A, Ozyurek H, Farin FM, Kroes HY, Wolfrum U, Brunner HG, Cremers FP, Glass IA, Knoers NV, Roepman R (2007) Mutations in the gene encoding the basal body protein RPGRIP1L, a nephrocystin-4 interactor, cause Joubert syndrome. Nat Genet 39: 882-888.

55. Satouh Y, Padma P, Toda T, Satoh N, Ide H, Inaba K (2005) Molecular characterization of radial spoke subcomplex containing radial spoke protein 3 and heat shock protein 40 in sperm flagella of the ascidian Ciona intestinalis. Mol Biol Cell 16: 626-636.

56. Kujala M, Hihnala S, Tienari J, Kaunisto K, Hastbacka J, Holmberg C, Kere J, Hoglund P (2007) Expression of ion transport-associated proteins in human efferent and epididymal ducts. Reproduction 133: 775-784.

57. Lonergan KM, Chari R, Deleeuw RJ, Shadeo A, Chi B, Tsao MS, Jones S, Marra M, Ling V, Ng R, MacAulay C, Lam S, Lam WL (2006) Identification of novel lung genes in bronchial epithelium by serial analysis of gene expression. Am J Respir Cell Mol Biol 35: 651-661.

58. Neesen J, Drenckhahn JD, Tiede S, Burfeind P, Grzmil M, Konietzko J, Dixkens C, Kreutzberger J, Laccone F, Omran H (2002) Identification of the human ortholog of the t-complex-encoded protein TCTE3 and evaluation as a candidate gene for primary ciliary dyskinesia. Cytogenet Genome Res 98: 38-44.

59. Iguchi N, Tanaka H, Nakamura Y, Nozaki M, Fujiwara T, Nishimune Y (2002) Cloning and characterization of the human tektin-t gene. Mol Hum Reprod 8: 525-530.

60. Tanaka H, Iguchi N, Toyama Y, Kitamura K, Takahashi T, Kaseda K, Maekawa M, Nishimune Y (2004) Mice deficient in the axonemal protein Tektin-t exhibit male infertility and immotile-cilium syndrome due to impaired inner arm dynein function. Mol Cell Biol 24: 7958-7964.

61. Roy A, Yan W, Burns KH, Matzuk MM (2004) Tektin3 encodes an evolutionarily conserved putative testicular microtubules-related protein expressed preferentially in male germ cells. Mol Reprod Dev 67: 295-302.

62. Hammond JW, Cai D, Verhey KJ (2008) Tubulin modifications and their cellular functions. Curr Opin Cell Biol 20: 71-76.

63. Mayer U, Ungerer N, Klimmeck D, Warnken U, Schnolzer M, Frings S, Mohrlen F (2008) Proteomic analysis of a membrane preparation from rat olfactory sensory cilia. Chem Senses 33: 145-162.

64. Santos N, Reiter JF (2008) Building it up and taking it down: the regulation of vertebrate ciliogenesis. Dev Dyn 237: 1972-1981.
